# Epigenetic Natural Variation in Arabidopsis thaliana

**DOI:** 10.1371/journal.pbio.0050174

**Published:** 2007-06-19

**Authors:** Matthew W Vaughn, Miloš Tanurdžić, Zachary Lippman, Hongmei Jiang, Robert Carrasquillo, Pablo D Rabinowicz, Neilay Dedhia, W. Richard McCombie, Nicolas Agier, Agnès Bulski, Vincent Colot, R.W Doerge, Robert A Martienssen

**Affiliations:** 1 Cold Spring Harbor Laboratory, Cold Spring Harbor, New York, United States of America; 2 Department of Statistics, Purdue University, West Lafayette, Indiana, United States of America; 3 Unité de Recherche en Génomique Végétale, CNRS UMR8114, INRA UMR1165, Université d'Evry Val d'Essonne, Evry, France; Oregon State University, United States of America

## Abstract

Cytosine methylation of repetitive sequences is widespread in plant genomes, occurring in both symmetric (CpG and CpNpG) as well as asymmetric sequence contexts. We used the methylation-dependent restriction enzyme McrBC to profile methylated DNA using tiling microarrays of *Arabidopsis* Chromosome 4 in two distinct ecotypes, Columbia and Landsberg *erecta*. We also used comparative genome hybridization to profile copy number polymorphisms. Repeated sequences and transposable elements (TEs), especially long terminal repeat retrotransposons, are densely methylated, but one third of genes also have low but detectable methylation in their transcribed regions. While TEs are almost always methylated, genic methylation is highly polymorphic, with half of all methylated genes being methylated in only one of the two ecotypes. A survey of loci in 96 *Arabidopsis* accessions revealed a similar degree of methylation polymorphism. Within-gene methylation is heritable, but is lost at a high frequency in segregating *F*_2_ families. Promoter methylation is rare, and gene expression is not generally affected by differences in DNA methylation. Small interfering RNA are preferentially associated with methylated TEs, but not with methylated genes, indicating that most genic methylation is not guided by small interfering RNA. This may account for the instability of gene methylation, if occasional failure of maintenance methylation cannot be restored by other means.

## Author Summary

In plants and animals, many DNA sequences are modified by the addition of methyl groups, but the principles governing methylation patterns are not well understood. In *Arabidopsis,* we show that repetitive sequences, derived from mobile (transposable) elements, are densely methylated throughout their length, while about one third of all protein-coding genes are internally methylated. Methylated transposons are silent, homologous to small interfering RNA, and coated with histone H3 dimethylated on lysine-9. In contrast, methylated coding-sequence genes are highly expressed, do not have corresponding small RNAs, and are coated with histone H3 dimethylated on lysine-4. Comparing two different ecotypes of *Arabidopsis,* we find that transposons are twice as likely as genes to have suffered insertion and deletion, although gene deletion is surprisingly prevalent. While the pattern of transposon methylation is conserved between ecotypes, protein-coding gene methylation is polymorphic so that only half of all gene methylation on any one chromosome is shared between natural accessions collected from around the world.

## Abstract 3

Two ecotypes of*Arabidopis* show different patterns of DNA methylation, which is heritable. Interestingly, differences in DNA methylation are not reflected in differences in gene expression.

## Introduction

In eukaryotic genomes, patterns of cytosine methylation are inherited from cell to cell through the action of maintenance methyltransferase enzymes on symmetrical CG or CNG dinucleotide pairs. Maintenance is not perfect, resulting in occasional loss of methylation from one sister chromatid, following replication of hemimethylated DNA. However, other methyltransferases can restore methylation patterns de novo, though the targeting mechanism is unclear [1,2]. While the function of DNA methylation is still debated, it is clear that methylation of promoter and enhancer regions can repress gene activity by preventing transcription-factor binding, as well as via histone modifying enzymes recruited by methyl DNA binding proteins [3].

In mammalian cells, CG islands in promoter regions remain unmethylated throughout development, allowing gene expression to be regulated by transcription factors in response to environmental and developmental signals [3]. Important exceptions include the Hox cluster genes, in which cytosine methylation may contribute to a “developmental memory” of gene regulation in the embryo, cooperating with histone methylation mediated by the polycomb complex [4,5]. Familiar examples of silenced genes associated with DNA methylation in mammals are imprinted genes and those found on the inactive X chromosome in females [3,5]. Similarly, in plants, genes in the endosperm, a terminal lineage, undergo changes in methylation as well as activation of the polycomb complex through imprinting [6]. Despite these examples, developmental regulation through DNA methylation and demethylation has proved elusive. The flowering time gene *FLC,* for example, is regulated by polycomb-mediated histone methylation but not by DNA methylation [7], while heritable paramutation at the maize *b1* locus depends on RNA dependent RNA polymerase but is not associated with major changes in DNA methylation [8,9]. Anecdotal evidence for heritable methylation polymorphism has been reported in plants and humans [10,11].

Transposable elements (TEs) carry protein-coding genes, but are usually [12], though not always, silent throughout development [13,14]. In plants, silent TEs are inherited from one generation to the next and are heavily methylated relative to genes. TEs are also methylated in vertebrate genomes but are not distinguished from most exons in this respect [15]. Also in vertebrates, methylation is lost during pre-implantation development and has to be regained during embryogenesis [3]. In both plants and vertebrates active transposons can regulate nearby genes. In this way mechanisms that silence transposons, such as RNA interference, histone modification, and DNA methylation, can also regulate genes [16,17].

In mammalian cells maintenance and de novo methyltransferase mutants are lethal, perhaps reflecting a large number of genes that are misregulated, for example by imprinting [3]. In plants there are multiple de novo methyltransferase genes *(DRM1* and *DRM2)* and a CNG methyltransferase *(CMT3),* but even double and triple mutants with the CG maintenance methyltransferase *(MET1)* are viable, though sterile [18,19]. Transposons are strongly activated in the most methylation-deficient double mutant strains [18,20,21]. In hypomorphs of *met1,* most developmental abnormalities are sporadic and irreversible following segregation of *met1* and include *superman* and *agamous,* which gain rather than lose DNA methylation for unknown reasons [2]. This is even more pronounced in the SWI2/SNF2 chromatin remodeling mutant *decrease in DNA methylation 1* (*ddm1*), which has no phenotype at first but gradually accumulates developmental abnormalities, transposon insertions, and infertility in subsequent generations [22,23]. In contrast, *drm/cmt3* [24] and *met1/cmt3* [25] double mutants have severe phenotypes, and defects in *met1/cmt3* double mutants are accompanied by increased expression of at least one gene required for embryonic development *(YODA),* although this gene is still expressed in wild-type (WT) embryos when it is methylated [19]).

The role of DNA methylation in gene expression has been examined using microarray profiles of DNA methylation mutants [17,26–28], but in these mutants it is mostly TEs (and genes under their direct control) that change in expression, rather than genes. As an alternative, we have used natural accessions of *Arabidopsis,* as opposed to mutants, to assess the extent and potential impact of DNA methylation. We employed a genomic tiling array of *Arabidopsis* Chromosome 4 that includes TEs, repeats, and genes to generate methylation profiles for Columbia (Col)-0 and Landsberg *erecta* (Ler) ecotypes, and found that, while TEs are heavily methylated in both ecotypes, genes are generally methylated only in parts of the coding region [29]. Furthermore, differences in gene methylation between ecotypes are common and heritable, but do not correspond to differences in gene expression. Finally, genic methylation is extremely polymorphic among 96 natural variation accessions, and the patterns of methylation are uncoupled from kinship-based phylogeny. We propose a model to account for these differences based on the differing roles of maintenance and de novo methylation.

## Results

### Comparative Genome Hybridization of Col and Ler

**Figure 1:**
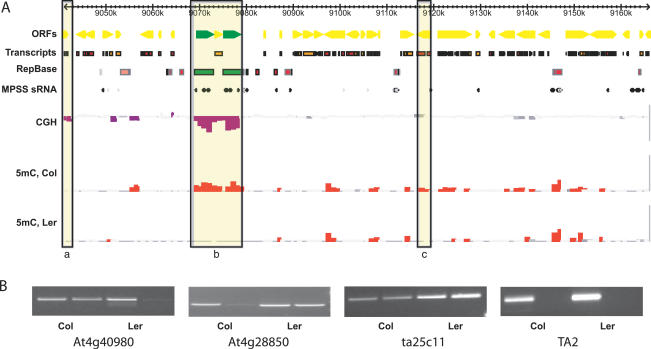
Methylation Profiles for Col and Ler *Arabidopsis* Ecotypes (A) Microarray data from Chromosome 4 are displayed for a 125-kb region 9 Mb from the nuclear organizing region (Figure S1). Open reading frames from genes (yellow) and retrotransposons (green) are indicated, along with repeats predicted by RepBase and TandemRepeatFinder. Small RNA matches from massively parallel signature-sequencing (MPSS) data are indicated. Tiles that represent significant CNPs are highlighted in purple (CGH), while tiles that detect significant DNA methylation are highlighted in red for the two ecotypes (5 mC, Col and Ler). Examples of a gene CNP (a), a TE CNP (b), and a methylation polymorphism (c) are boxed. (B) Significant methylation was detected by microarray analysis for two representative genes in Ler *(At4g40980)* and Col *(At4g28850),* respectively. This methylation was verified by digestion of genomic DNA by McrBC, followed by PCR amplification using primers specific for each gene (lower panels). Failure to amplify a product after digestion by McrBC indicates that the gene is methylated. Control primers from an unmethylated tile (ta25c11) and a methylated transposon (TA2) indicate complete digestion and amplification in each case.

Methylation profiles for Ler and Col were determined using McrBC digestion and size fractionation as previously described [30], except that the tiling microarray was derived from the entire sequence of Chromosome 4 [31]. This array, described in detail previously [31], comprised 21,815 printed tiles, each consisting of an approximately 1-kb PCR product amplified with sequential primer pairs along Chromosome 4. Single copy regions were represented on their own as much as possible [31]. Genomic DNA samples were prepared from independent batches of 14-day-old seedlings to match as closely as possible methylation and expression profiles from smaller arrays covering part of the short arm of Chromosome 4 [17]. A total of three biological replicates (independent batches of seedlings) were performed for each genotype using dye swaps (technical replicates), and hybridization intensities were normalized using a linear model to partition the variance and estimate experimental error (Materials and Methods). Tiles with significant ratios of digested to undigested DNA were detected by comparison against 576 intergenic tiles without repeats (Materials and Methods). The resulting profiles were displayed as histograms aligned with the chromosome sequence, annotation, and other features of interest, using a customized implementation of the Generic Genome Browser (http://www.gmod.org). For the purposes of analysis, microarray tiles were classified as matching genes, TEs, tandem repeats, or genes within internal repeats, or as having no annotation. The TE and repeat annotation used in this study combines the most current *Arabidopsis* Information Resource (TAIR) (http://www.arabidopsis.org) genome release, a TandemRepeatsFinder analysis, and sensitive CENSOR-based identification of TE homologies. Because even small regions of TE similarity or repeat content might induce heterochromatic modifications, we classified tiles as TE or repeat if 5% of the tile matched these entities. The methylation profiles for Ler and Col were remarkably similar, with strong uniform signals detected in heterochromatin (Figure S1). As previously reported [17,26–28], lower levels of methylation were also detected in euchromatin, but many of methylated loci differed between Ler and Col (Figure 1).

Some differences between Ler and Col methylation profiles could be due to simple copy number polymorphisms (CNPs) between the two genomes. To address this possibility as well as to investigate the degree of gross interecotype genetic diversity, we performed comparative genome hybridization (CGH) with the same non McrBC-digested genomic DNA samples used in the methylation profile. Significant CNPs were detected using linear model analysis of variance. Because the array design is based on the sequence of Col, only decreases in the Ler genome could be mapped, although amplifications elsewhere in the genome could also be detected. Decreased copy number in Col relative to Ler could not be identified, nor could simple rearrangements. In Ler, we found 27 of the 36 Chromosome 4-specific deletions reported previously in the CGH analysis of Borevitz et al. (Table S1) [32]. We compared our CGH data to sample sequences from shotgun sequencing of the Ler genome, which represent approximately 60% nucleotide coverage of that genome [33], and we found that less than 15% of the tiles with decreased copy number in Ler matched sequence reads over 60% of their length, while 50% of all non-CNP tiles matched at this level of coverage, suggesting that most of the Ler-specific CNPs detected by this microarray analysis were real. Furthermore, comparison of our CGH data at the RPP5 locus, which has been sequenced in its entirety in Ler, showed that we were able to identify fine-scale CNPs as well as larger deletions (Figure S2).

We found that over 10% of tiles corresponding to TEs and repeats were missing from available Ler sequence (Table 1). This was not unexpected, as TEs were already known to be polymorphic [32,34,35]. More surprisingly, we found that 504 tiles representing 390 distinct genes in Col are at least partially deleted from Ler (or are extremely divergent in sequence). Most of these genes were not duplicated elsewhere in the genome and were thus unlikely to be pseudogenes (Table S1). Assuming the same degree of CNP in Col and extrapolating to the whole genome, more than 2,000 genes may be at least partially deleted in one or the other ecotype. Over half of these genes are expressed (see below), indicating that many, though certainly not all, of them are functional. Extensive interindividual CNP has been identified in humans and is hypothesized to play a substantial role in spontaneous genetic disease [36]. Thus, the observed *Arabidopsis* CNPs are expected to provide a rich source of candidate genes for quantitative trait loci [13,32].

**Table 1:**
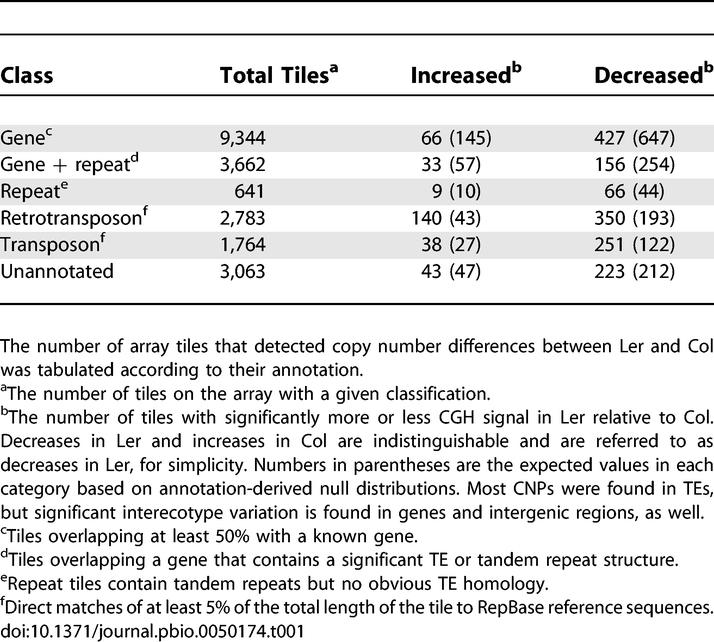
CNP between *Arabidopsis* Ecotypes

### Methylation within Genes Is Localized and Polymorphic

**Figure 2:**
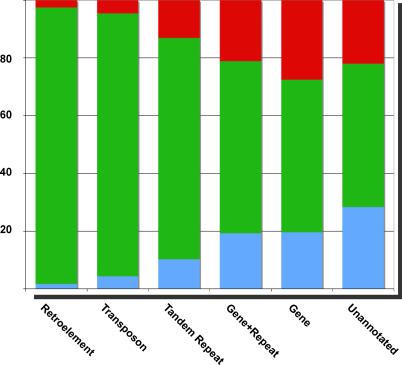
Variation in Methylation of Genes and Transposons Methylation status of tiles detecting significant methylation but not overlapping CNPs (Table 1) was compared between ecotypes. Among these were tiles annotated as retrotransposons (1,551), transposons (623), tandem repeats (107), genes with repeats (904), genes (2,174), and unannotated (304). Methylation in Col (red), both ecotypes (green), and Ler (blue) is represented in the proportional bar graphs for each class of tile. A total of 45% of genic and unannotated tiles that detected significant methylation did so in only one of the two ecotypes, while 94% of TE tiles detected significant methylation in both (Table 2).

We examined the distribution of methylation patterns within the genome by comparing array features between ecotypes according to their methylation status and their annotation. We found that, as expected, tiles annotated as TEs were more likely to be methylated (Table 2), such that 85% of annotated TEs had at least one methylated tile (Table 3). TEs were uniformly methylated in Col and Ler, with fewer than 9% of class II TEs and 4% of class I TEs differing in methylation between ecotypes (Figure 2). However, in agreement with recent studies [17,26–28], approximately 21% of gene tiles (corresponding to one-third of genes) were also at least partially methylated (Table 2). In contrast to TEs and repeats, methylation in genes was highly variable (Figure 2), with approximately 50% of methylated tiles differing between the two ecotypes. Some methylation profile variants corresponded to CNPs in one or other ecotype, such that the observed difference in methylation was likely to be a consequence of CNP (Figure 1). However, the vast majority of the variation detected by methylation profiling was not due to CNP, and the variation was very widespread.

**Table 2:**
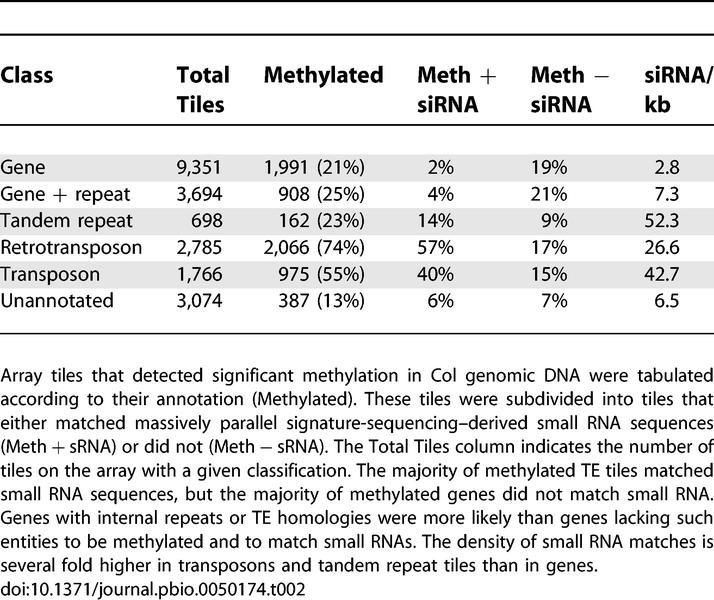
Summary of Methylated Features

**Table 3:**
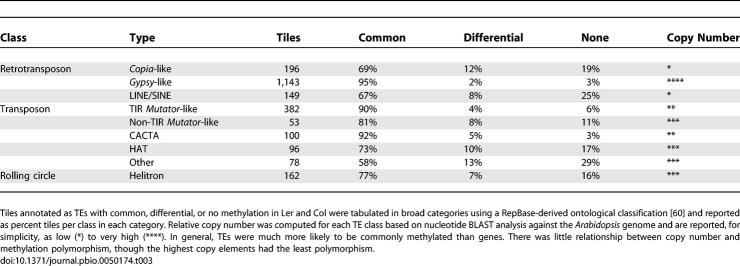
Methylation Polymorphism at TEs

Substantial replication was used to provide high confidence in both methylation profiles, and approximately 80% of methylated genic tiles previously detected in Col on the short arm of Chromosome 4 [17] were found to be methylated in the present study (unpublished data). To further investigate the validity of our methylation profiles, we surveyed a sample of 28 tiles in each ecotype using digestion with McrBC followed by amplification by PCR [15,17], comparing each result to the prediction from our microarray analysis. Methylation status of 50 of the 56 tiles (90%) matched the microarray (Table S2). The six remaining tiles in Col had low levels of methylation detected on the array, the nature of which is described further below.

**Figure 3:**
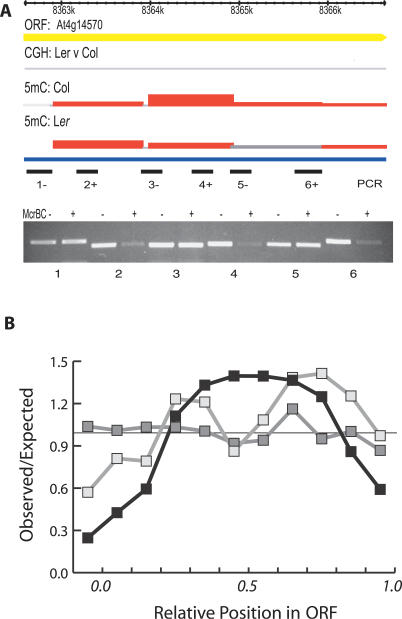
Methylation is Localized within Genes (A) Intermediate levels of methylation were detected by microarray analysis in Col and Ler for one representative gene (genome browser tracks are annotated as in Figure 1). Primer pairs 1–6 are indicated below each tile of the array. Amplification of McrBC digested Col DNA (+) and undigested Col DNA (−) was performed as in Figure 1. Failure to amplify digested DNA indicated that methylation was localized to regions 2, 4, and 6, rather than spanning the entire gene. (B) The number of genic tiles detecting significant methylation was calculated at 10% intervals relative to the length of each gene and compared with the number expected if methylation was randomly distributed (black line). Only genes larger than 2 kb (three tiles or more per gene) were considered. Methylated tiles differing between ecotypes were also plotted in a similar way (light gray line). Genic methylation is largely concentrated in the middle of genes. For comparison, methylation distribution as a function of position was also calculated for TE-derived open reading frames (dark grey line) and is uniformly distributed.

Given that our results represent quantitative measures of DNA methylation, on average, TEs had four to seven times more methylation signal per kb than genes (Figure 1 and unpublished data). Since total input DNA is used to control each hybridization, signal strength depends on the extent of McrBC digestion in the depleted sample rather than the tile's copy number in the genome. If they are unmethylated, high copy-number sequences contribute the same signal strength to both fluorescence channels so that the ratio is still close to 1.0 [37]. Short methylated regions occupy only part of a 1-kb tile and permit McrBC digestion of only some of the target DNA fragments that hybridize with the tile; target DNA fragments located outside the methylated region remain undigested and still hybridize. Complete methylation of the tile, on the other hand, would result in complete loss of target fragments on digestion with McrBC. Intermediate signals would result from multiple, short regions of methylation within the tile. We tested this idea on tiles with microarray signals of intermediate strength. PCR was performed on McrBC-digested genomic DNA using a series of internal primer pairs corresponding to each tile. As predicted, methylation was only detected with three of the six primer pairs tested, indicating that only short regions within each tile were methylated (Figure 3A). Similar “clusters” of methylated CpG dinucleotides were previously detected by microarray profiling and bisulphite sequencing in a sample of 20 *Arabidopsis* genes [26] and are suggested by results of other recent methylation profiling studies [27,28].

Next, we examined the physical distribution of significantly methylated tiles within genes. During the design of the microarray, tile positions were chosen independently of gene annotation, and thus the 9,344 tiles annotated as “gene” are distributed randomly across 3,830 Chromosome 4 genes. We used this fact in an average gene analysis to examine methylation patterns in genes represented by multiple tiles and having no annotated repeats. By comparing the frequencies of significantly methylated tiles observed at a given position with those expected for a random distribution across genes, we found that the middle of genes was enriched for methylation relative to the promoters, first exons, and 3′ ends, in agreement with other studies (Figure 3B) [17,26–28]. When the analysis was further restricted to tiles that differed in methylation between Col and Ler, the distribution was the same (Figure 3B), indicating no positional bias for methylation polymorphisms. In contrast, methylation was evenly distributed across TE-derived open reading frames (Figure 3B), suggesting that the mechanism of TE methylation is distinct.

We considered several possible explanations for the hypervariable nature of genic methylation relative to TEs (Figure 2). First of all, many TEs have a higher copy number than genes, so that loss of methylation from only one copy might go undetected. In support of this idea, very high copy gypsy-like retrotransposons were the least polymorphic, while low copy copia-like retrotransposons and class II Ac-like HAT transposons were the most polymorphic with respect to methylation (Table 3). However, unmethylated elements could be detected in all classes of TEs, and genes were more likely to vary in methylation than even low copy TEs (Table 3), so that copy number alone cannot account for the lower variability in TEs. Another possibility was that genes might lose methylation more readily than TEs, because they were less heavily methylated to begin with and were not targeted by mechanisms that guide de novo methylation. We tested this idea by examining stability of genic methylation patterns over multiple generations.

**Figure 4:**
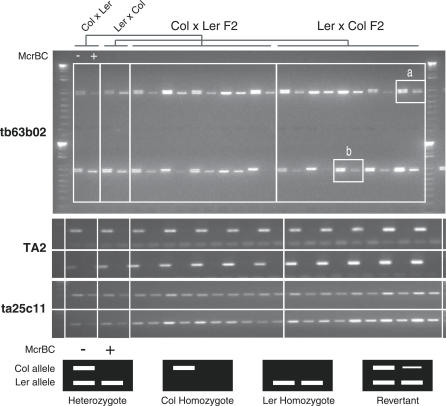
Heritability of Polymorphic Gene Methylation The gene *At4g28850* is methylated in Col but not Ler. Genomic DNA was prepared from *F*_1_ and *F*_2_ siblings derived from reciprocal crosses between Col and Ler and subjected to McrBC digestion and PCR amplification of this locus as in Figure 1. The amplification product (tb63b02) has a small deletion in Ler, enabling the parental alleles to be distinguished. DNA samples were digested (+) or mock-digested (−) with McrBC. Control primers were used to amplify a methylated retrotransposon (TA2) in each sample, as well as an unmethylated control tile (ta25c11). Samples of two *F*_1_ and ten *F*_2_ plants are shown for each cross (out of a total of eight and 40, respectively) (Table S3). In almost all cases, the Col allele is digested by McrBC, and the Ler allele is never digested. Two exceptions are indicated (a and b) in which the Col allele has lost all or most of its associated methylation.

The heritability of genic methylation in *F*_2_ families was tested using the previously described PCR assay. We chose two genes *At4g28850* and *At4g18020*, which were methylated in Col but not in Ler and also had a DNA sequence polymorphism (either a single nucleotide polymorphism or an indel), allowing parental alleles to be distinguished. Genomic DNA was prepared from 16 *F*_1_ hybrid plants generated by reciprocal crosses between Col and Ler, as well as from 80 of their *F*_2_ progeny. The DNA was digested with McrBC and then amplified by PCR using primers specific for each gene, as well as from control tiles. Infrequent loss of parental methylation was detectable in the *F*_1_ generation, so that 15 of 16 plants inherited methylation at *At4g28850,* and 14 of 16 inherited it at *At4g18020* (Table S3). In the *F*_2_, 54 of 62 plants inheriting a Col allele of *At4g28850* also inherited methylation (Figure 4; Table S3), while 56 out of 59 plants inherited methylation at *At4g18020*. In the Col × Ler cross, the *F*_1_ plants did not lose methylation at *At4g18020* nor did their *F*_2_ progeny (Table S3). This suggests that methylation was lost during development of *F*_1_ hybrids and that state was inherited in the *F*_2_ progeny. Genic methylation is therefore heritable but unstable, and such instability likely accounts for the extreme polymorphism observed between ecotypes. The unmethylated state of the parental Ler allele is inherited faithfully at both genes through two generations, indicating that, in spite of obvious interallelic sequence homology, methylation is not propagated in *trans* from methylated Col alleles (Table S3). However, small quantitative differences that would arise from sporadic de novo methylation of individual cytosines might have gone undetected in our assay.

### Mechanism and Consequences of Genic Methylation

Sampling of methylated genes has previously indicated that genic methylation is predominantly localized in clusters of CpG dinucleotides [26,38]. Consistent with these data, genic methylation is under the control of *MET1,* but is relatively unaffected in mutants in *DDM1,* while TEs lose methylation in both mutant backgrounds [17,27]. This might account for the differential polymorphism of genes and TEs, if TEs are actively targeted for methylation by *DDM1, DRM1/2,* and *CMT3*, as well as by *MET1* [17,26,27]. One idea is that this targeting is accomplished via small RNAs, large numbers of which correspond to TEs [12,39]. However, small RNA have also been proposed to guide gene methylation. *PHABULOSA,* which is a microRNA target, is methylated in a short region located downstream of the ath-miR165/166 recognition site, which lies, quite unusually, at a splice junction [38]. It has been proposed that this arrangement results in recruitment of methylation to the gene by way of the spliced nascent transcript binding microRNA. It has similarly been proposed that small interfering RNA (siRNA) may guide methylation of cryptic initiation sites within transcriptional units that otherwise reduce transcription levels, although the extent of this reduction is barely detectable [28].

A large number of small RNAs have been sequenced from *Arabidopsis,* representing a significant fraction of the total siRNA population [40]. We analyzed methylated tiles from the microarray for matches to siRNA (Table 2). While 71%–76% of methylated tiles annotated as TEs also corresponded to siRNA sequences, as previously reported [17,27], only 11% of methylated gene tiles correspond to siRNA (Table 2). Conversely, only 96 of 258 (37%) genes that matched siRNA anywhere along their length had at least one methylated tile, compared with 32% of all genes, the vast majority of which do not match siRNA. The slight difference reflects a handful of genes with large numbers of siRNA that may either be novel TEs or contain unidentified repeats. Thus, if siRNAs direct methylation, their role is restricted almost entirely to TEs. Further support for this idea comes from the observation that, *ATHB8,* a Chromosome 4 homolog of *PHABULOSA,* which also matches ath-miR165/166, does not have detectable methylation in either Ler or Col. Furthermore, genic methylation does not correspond to heterochromatic marks such as H3K9me2 [17] nor is it dependent upon *DRM1/2* or *CMT3,* which is thought to associate with siRNAs as part of the methylation pathway [27].

To test whether CNPs and genic methylation might affect gene expression, we compared our profiling data to Affymetrix *ATH1* expression profiles for Ler and Col seedlings [41]. When we mapped *ATH1* probe sets to features on our Chromosome 4 tiling array, we found that 3,292 of 3,982 genes were represented. Based on our CGH data, 88 of these genes were predicted to have decreased copy number in Ler and thus might have altered expression in Ler relative to Col. For three genes, decreased Ler copy number resulted in significantly lower expression (Table S4). In most of the remaining cases, the CNPs did not overlap with all the available *ATH1* probes in a given probeset. That expression signals were unchanged in these genes relative to Col suggests that many of the CNPs we identified represent alterations in gene structure rather than full-scale deletions. Interestingly, expression of the gene *At4g04330* was significantly higher in Ler even though it was predicted to have a deletion in that genotype. Closer examination revealed the presence of a HAT transposon inserted into the largest intron that is absent in the Ler genome sequence. Presumably, this transposon mitigates expression of the Col gene. We next examined the consequence of methylation upon 1,981 of the 3,292 genes that had no repeats or annotated TE fragment. A total of 520 of these genes had a least one methylated tile in both ecotypes, while approximately 317 had methylated tiles in one ecotype but not in the other. Approximately 6% (19/317) of differentially methylated genes had significantly different expression between ecotypes, indicating potential regulation, but a similar number of unmethylated genes were also differentially expressed (3%) (Table S5). In comparison, when all genes on the *ATH1* array are considered, around 6% of 22,810 genes have ecotype-specific expression, further suggesting that DNA methylation is not playing an active role in regulating gene expression. In addition, genes with detectable methylation in their promoter regions were no less likely to be expressed than those lacking methylation (unpublished data). We conclude that the low level of methylation found in genes does not lead to a general repression of gene expression, although individual genes may be regulated in this way [29]. In fact, in agreement with recent studies [28], expressed genes were actually more likely to be methylated than unexpressed genes (Table S5), as though expression might somehow direct genic methylation. This was in sharp contrast with methylation of TEs, which strongly associates with silencing [17].

**Figure 5:**
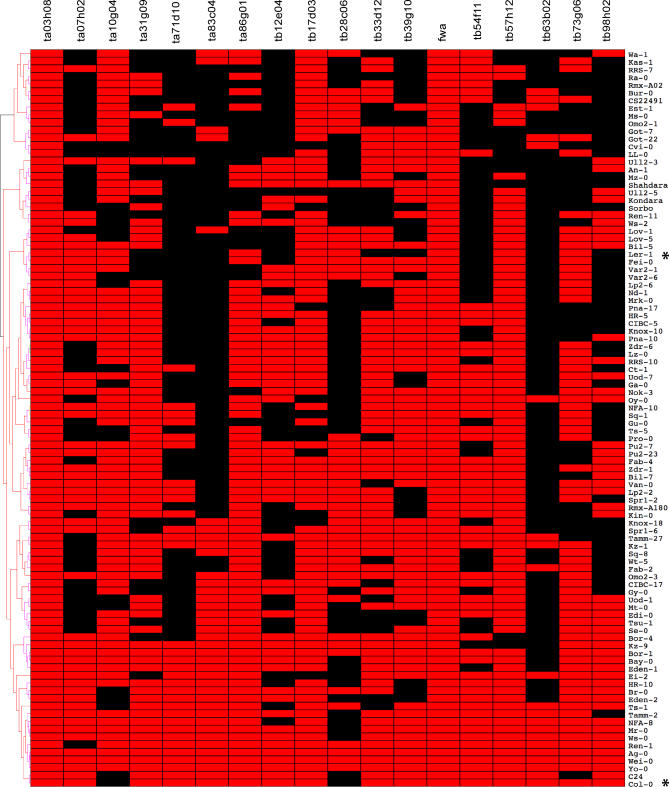
Genic Methylation among 96 *Arabidopsis* Accessions Methylation polymorphism was surveyed at 18 genomic loci in 96 natural variation accessions of *Arabidopsis* [43], including the flowering time control locus *fwa*. Equal amounts of undigested (McrBC−) and digested genomic DNA (McrBC+) from each accession were amplified using PCR with primers specific for each locus. Methylation was scored as binary traits, represented in the graphical matrix by red (methylated) or black (unmethylated). Col-0 and Ler-1 are highlighted with an asterisk, and loci are arranged left to right in correspondence with their physical order on Chromosome 4. Per-accession methylation profiles were clustered using unweighted pair group method with arithmetic mean, and a support tree was generated using 10,000 bootstrapped replicates. The resulting tree bears no resemblance to a recent kinship-based phylogeny [42], and no major branches of the tree have significant support (unpublished data).

Col and Ler gene methylation was highly divergent, consistent with the relatively large evolutionary distance between these ecotypes [42]. To obtain a more comprehensive portrait of interecotype epigenetic diversity, we selected 18 loci (Table S9) methylated in Col and/or Ler for further analysis in 96 accessions [43] using McrBC digestion and PCR to detect methylation (Materials and Methods). A visual summary of our results can be found in Figure 5. We found that genes methylated in Ler, Col, or both ecotypes were also methylated in some, but not all other accessions, while genes unmethylated in Ler and Col were methylated in at least some other accessions. In contrast, the low-copy *TA2* copia-like transposon was methylated in all accessions tested (Figure S3). Interestingly, closely related accessions, such as TAMM2 and TAMM27, were no more likely to share methylation patterns than distantly related ones, such as Mr-0 (Italy) and HR5 (United Kingdom). When the methylation patterns are clustered using hierarchical clustering (Figure 5), the resulting tree bears little or no resemblance to one based on kinship, as measured by pairwise haplotype sharing [42].

Nearly all genes surveyed exhibited substantial methylation polymorphism, with notable exceptions of *FWA* and *At4g00500*. The latter encodes a lipase with no TE, repeat, or siRNA homology, and its uniform methylation across all accessions is unexplained. *FWA* is an imprinted gene on Chromosome 4, and methylation was detected in the first two non-coding exons in both Col and Ler, in agreement with previous results [44,45]. The first two exons comprise tandem repeats that are part of a SINE element integrated at the locus [17,46]. In this case DNA methylation and associated siRNA are critical for gene silencing and control of flowering time, and methylation is invariant among accessions (Figure 5). Thus while TE methylation is mostly invariant, genic methylation is highly variable.

## Discussion

Quantitative DNA methylation profiling of an entire plant chromosome has revealed that the majority of DNA methylation is found in TEs, which are methylated throughout their length and are up-regulated in *ddm1* and *met1* mutants due to the loss of cytosine methylation in these mutants [12,17]. Satellite repeats found in the heterochromatic knob on Chromosome 4, as well as in the inner pericentromeric repeats, are also methylated, but interestingly they have lower methylation signals than surrounding TEs (Figure S1; http://chromatin.cshl.edu/epivariation). Centromeric satellite repeats have 16–18 potential McrBC half sites per 180 bp, which is typical of the genome as a whole, so that this reduced signal presumably reflects the presence of unmethylated tracts of centromeric repeats.

This profiling also indicates that many genes are methylated, though at a substantially lower level than TEs. This genic methylation is typically found in internal regions not usually responsible for transcriptional regulation. These results are in agreement with previous studies indicating that one out of four of *Arabidopsis* genes on the short arm of Chromosome 4 are methylated [17] and that clusters of methylated cytosines occur within some transcription units [26]. Similarly, in maize, 5%–7% of exons are methylated, and most genes have four to five exons, so that 25% of genes have at least some methylation [15]. Our Col profile also agrees to a remarkable extent with recently published genome-wide profiles of *Arabidopsis* DNA methylation based on immunoprecipitation of methylated DNA with antimethylcytosine antibodies (Figure S4; Table S8) [27,28], considering the different methods employed. However, out of 1,728 genes in which some methylation was detected on Col Chromosome 4, 394 were only found in our dataset, compared to 151 and 103 genes uniquely identified by each of the other studies (Figure S5). This may reflect our analytical technique, as the other studies did not employ extensive statistical analysis [27] or replication (Protocol S1) [28].

By examining two different ecotypes, we show that a significant number of genes and TEs are methylated or altered in copy number between *Arabidopsis* ecotypes. TEs are more likely to be deleted than genes, but are heavily methylated in all ecotypes. On the other hand, genes have a lesser though significant degree of CNP but exhibit very high levels of epigenetic variation among ecotypes (Figure 5; Table 1). One explanation for this variation is that methylation of genes arises spontaneously, in conjunction with or as a consequence of transcription (Table S4) [27,28], and is then maintained imperfectly. Gene methylation is largely dependent on *MET1* [17,26], the maintenance methyltransferase responsible for methylation of CpG dinucleotides. It is thought that the mammalian homolog of *MET1*, *DNMT1*, maintains methylation at approximately 95%–99% of hemimethylated CG dinucleotides per cell division [47] and can also methylate about 3%–6% of unmethylated CpG dinucleotides de novo [48]. This could account for the instability of *MET1*-dependent genic methylation that we have detected (Figures 4 and 5), which itself is reminiscent of the instability typical of epimutant phenotypes [49,50]. While we failed to quantitatively detect the gain of methylation in two genes among 80 individuals, de novo methylation of genes over evolutionary time would account for the observed polymorphisms.

Epigenetic variation can, in principle, cover a range of relationships with the underlying genotype; from epigenetic variation associated with primary genetic polymorphisms to completely uncoupled genetic and epigenetic variation [51]. One gene we identified that was uniformly methylated in every ecotype is *FWA* (Figure 5), whose demethylation has important developmental consequences in flowering. In this case, the methylated exons are contributed by the insertion of a *SINE3* non-long terminal repeat retrotransposon. DNA methylation of *FWA* is guided by siRNA from this TE and is thus very stable [17,52]. TE methylation can be specifically targeted by chromatin remodeling and RNA interference, working through the methyltransferase genes *DRM1, DRM2,* and *CMT3,* which can quickly restore silencing if it is lost [39,53]. Genes, on the other hand, are not targeted by this pathway and lose methylation permanently if *MET1* fails to detect hemimethylated substrates following replication.

Zilberman et al. [28] propose that genic methylation functions to silence aberrant transcription from cryptic initiation sites that might be exposed during gene transcription. In this model, siRNA produced from aberrant transcripts recruits silencing machinery, including DNA methyltransferases, to the chromatin. Methylation of genes, however, is independent of *DDM1, DRM1, DRM2,* and *CMT3,* which are requisite components of the siRNA-mediated silencing pathway [26,27,53], and heterochromatic histone modifications such as H3K9me2 do not associate with regions of genic methylation [17]. Recently, profiles of H3K27me3, H3K27me2, H3K9me2, and H3K9me3 have been obtained using the same microarray platform, and none of these profiles overlapped significantly with genic methylation [54]. The primary basis for this model is a weak correlation between siRNAs and gene methylation (Table 2) [28], but this is probably due to unidentified TE fragments or repeat structure within some methylated genes that can directly recruit the heterochromatic silencing apparatus (Protocol S1). An additional argument against involvement of siRNA in genic methylation comes from our inheritance studies, which showed that, in Col-Ler hybrids, unmethylated Ler alleles of *At4g28850* and *At4g18020* did not gain methylation in *trans* from the Col alleles (Figure 4; Table S3).

In contrast to *FWA*, methylation of genes only rarely corresponds to changes in gene expression. Our results are in agreement with the recently published genome-wide methylation and transcriptional profiles of the Col ecotype. In one study, ~5% of methylated genes and pseudogenes were differentially expressed in *met1–3* mutants in which nearly all genic methylation was lost [27], and in the other, methylated genes were only 14% more up-regulated in *met1–6* than were unmethylated genes [28]. These findings are interpreted to indicate either that expression of substantial number of genes is under direct control of DNA methylation [27] or that genic methylation exacts a cost in the form of decreased transcription elongation efficiency [28]. However, in both cases, many of the open reading frames and pseudogenes considered to represent genes contained substantial TE homology or repeat structure and were thus likely to behave as such in transcription studies in a *met1* background (Protocol S1). We found that only 6% of methylated genes were differentially expressed after loss of DNA methylation in one or other ecotype (Table S5) and that unmethylated genes were just as likely to be differentially expressed between ecotypes. The possibility remains that methylation can influence gene expression in only a few cells, so that differences between ecotypes and mutants are too small to detect. Also, expressed genes were generally more likely to be methylated than unexpressed genes (Table S5), and methylated genes are nearly twice as likely to be associated with H3K4me2 than unmethylated genes (not shown; http://chromatin.cshl.edu/epivariation/). Thus, it is possible that H3K4 methylation, rather than H3K9 or H3K27, guides DNA methylation of genes, but this awaits more rigorous investigation.

Along with loss of methylation from individual TEs, Zhang et al. also reported novel genic methylation in triple *drm1 drm2 cmt3* mutants relative to WT [27]. This would be inconsistent with the idea that methylation of TEs and genes is mediated by these enzymes [39]. On our microarrays, the majority of this novel methylation on Chromosome 4 was readily detected in WT Col plants (Protocol S1). This discrepancy may reflect technical failure to detect methylation in the WT sample or perhaps the instability of methylation patterns between the biological replicates used by Zhang et al. [27].

In conclusion, we propose that methylation of expressed genes occurs, but is also lost frequently, leading to the high levels of epigenetic diversity among populations. Gene methylation is not guided by siRNA or by microRNA, but is maintained by the DNA methyltransferase MET1, whose occasional failure to detect hemimethylated DNA following replication accounts for the observed instability. Methylation within genes generally has no effect on expression, unless it occurs in the promoter or other regulatory region, in which case it presumably becomes subject to and is generally lost via purifying selection. This could explain the depletion of methylation at the 5′ end of genes, and a similar case can be made for the 3′ end of genes, if, for example, methylation were to affect 3′ end processing. Gene methylation might still play a major role in evolution, given the high frequency with which it can arise [51] and revert when selection pressures change [55]. Methylation of TEs, on the other hand, is actively guided by de novo methyltransferases, histone modification, and siRNA, so that methylation patterns can be restored if they are inadvertently lost. TE methylation is therefore much more stable than gene methylation and can bring genes under control when TEs integrate nearby. Relaxation of this control would occur upon excision or deletion of the TE. Methylated TEs, such as the SINE element at *FWA* and methylated promoters (such as *MEDEA*) can be regulated during development via the action of DNA demethylases [45,56], though it remains to be seen how widespread this regulation might be.

## Materials and Methods

### DNA extraction, labeling, and array hybridization.

Col and Ler ecotypes of *Arabidopsis* plants were grown under long days in the greenhouse under standard growth conditions. Approximately 100 14-day-old seedlings were pooled together and used for DNA extraction as described previously [17] (within-ecotype epigenetic variation in *Arabidopsis* was estimated to be less than 1% [57]). DNA was digested with McrBC (New England Biolabs, http://www.neb.com), labeled with Cy3 or Cy5 dye, and hybridized to the Chromosome 4 tiling array as previously described [30,31]. For the CGH experiment, labeled DNA from Col and Ler ecotypes were hybridized on the same array, while for methylation profiling DNA from a single ecotype was hybridized to an array to allow for the comparison between untreated and McrBC-treated DNA. Using a dye swap experimental design (technical replicates), three biological replicates, from separate pools of seedlings, were performed for each ecotype; a total of six arrays were hybridized. An extended explanation of these protocols can be found in Protocol S1.

### Microarray analysis.

*Arabidopsis* Chromosome 4 tiling microarray was designed from the entire sequence of Chromosome 4 and comprised 21,815 printed tiles, each consisting of an approximately 1-kb PCR product amplified with sequential primer pairs along Chromosome 4. Over 50% of tiles represent single copy regions as identified by BLAST analysis of sequential 100-bp windows of sequence against the entire *Arabidopsis* genome sequence (Protocol S1) [31].

Complete details of microarray analysis and bioinformatic approaches can be found in the Protocol S1. In summary, both the CGH and methylation genomic tiling microarray data were analyzed separately using a linear model and ANOVA (for details see [31]). Specifically, the linear model ln(*Y*_ijkmr_) = μ + *A*_i_ + *D*_j_ + *T*_k_ + *G*_m_ + *AG*_im_ + *DG*_jm_ + *TG*_km_ + *ɛ*_ijkmr_ was employed to partition the sources of variation. The natural logarithm transformed, background corrected data are denoted as ln(*Y*_ijkmr_). The overall mean effect is μ*,* and *A, D, T,* and *G* represent the array, dye, treatment, and gene (or feature) main effects, respectively. The interactions of the main effects are AG, DG, and TG and represent array by gene, dye by gene, and treatment by gene, respectively. The random error *ɛ*_ijkmr_ is assumed to be normally distributed, with mean zero and constant variance. Once the sources of technical variation (e.g., global and feature-specific array and dye effects) and experimental variation (e.g., treatment and treatment by feature interaction) are estimated, differential fluorescence in the CGH experiment was tested using hypotheses that acknowledge both the average treatment effect and the treatment by feature interaction [58]: *H*_0_:*T*_k_ + *TG*_km_ = *T*_k'_ + TG_k'm_ versus H_a_:T_k_ + TG_km_ ≠ T_k'_ + TG_k'm_. Statistically significant differences were determined by evaluating signals at each tile as compared to the entire tiling array using a two-sided t-test. Alternatively, when testing for differential methylation it was necessary to use control features that enabled the detection of subtle signals coming from euchromatic methylation, as well as heterochromatin. To accomplish this, 576 randomly selected tiles across Chromosome 4 regions with no gene or repeat annotation were used as controls (Table S6). These controls supplied an average unmethylated value against which other features on the chromosome were tested using a one-sided t-test. For both the CGH and methylation experiments, the multiple testing issues that arise when testing 21,815 tiles across Chromosome 4 were addressed by controlling the false discovery rate (FDR) at the 5% level using a Benjamini and Hochberg correction.

Methylation and CGH profiles were loaded into a MySQL relational database implementing the Bio::DG::GFF schema, thus facilitating intersecting positional, quantitative, and class-based queries and computations. In addition, array data and genome annotations were displayed visually using a Generic Genome Browser, available for public examination at http://chromatin.cshl.edu/epivariation.

Affymetrix CEL files for Col and Ler expression profiles were obtained from TAIR (AtGenExpress, ten ecotypes in triplicate). Normalized expression estimates were calculated using the gcRMA algorithm (http://www.bioconductor.org). Per-ecotype probe set values and significant interecotype differences were computed using the limma package in R, with FDR controlled at 0.05 [59]. A gene was considered to be expressed in a given ecotype if its normalized value fell into the upper mode of the bimodal distribution of all expression values for that ecotype. Probe set sequences for *ATH1* were obtained from Affymetrix and positioned on the genome using BLAT. These genomic positions were then interpolated to correspondence with specific tiles on the Chromosome 4 array.

###  McrBC PCR.

McrBC PCR confirmation of genic methylation identified via microarray analysis was performed on genomic DNA that was extracted from 14-day-old rosette leaves from a pool of a dozen plants grown under identical conditions as described above. This DNA was treated with McrBC in the same manner, and PCR primers used to amplify microarray tiles from *Arabidopsis* genomic DNA (Table S7). For McrBC PCR of *F*_2_ plants from a cross between the Col and Ler ecotypes, as well as the 94 other accessions from the Nordborg et al. [43] collection, DNA was isolated from a pool of rosette leaves of a dozen plants of identical age as above, and 10 μg of DNA was digested with 10 U of McrBC for 8 h. We used 4 μl of DNA from digested and mock-digested DNA as a template in a 20-μl PCR reaction with 24 cycles of amplification for each primer pair. Primer sequences used in various PCR reactions are listed in Table S3.

## Supporting Information

Figure S1 Chromosome-Scale Comparison of Col and Ler Methylation Profiles Microarray data from the first 9 Mb of Chromosome 4 are displayed as in Figure 1. Cytological heterochromatin is indicated by the dark purple bars underneath the browser map and corresponds to the most densely methylated region of the chromosome. Individual methylated transposons can also be distinguished as major peaks on the chromosome arms, corresponding to green (retrotransposons) and red (transposons) features, respectively, in the annotation track. Genes are indicated as yellow features in the annotation track. Small RNAs are indicated by blue tick marks below the gene and repeat tracks.
(93 KB PDF)

Figure S2 Comparison of CGH Data to Known Genomic Sequence at the RPP5 Locus Microarray-based CGH results are compared to the Ler genomic sequence for the RPP5 disease resistance locus [61] using BLASTN. Significant Col-Ler high-scoring pairs and their percent identities are displayed as a green bar graph. Significant CNPs in Ler versus Col are detected as a decline in BLASTN percent identity or significant CGH differences. The two datasets show remarkable agreement in both TE and gene domains of RPP5.
(23 KB PDF)

Figure S3 Control McrBC PCR A total of 96 natural variation accessions were digested with McrBC, and the undigested and digested samples were used as templates in PCR reactions with primers specific for an unmethylated region as the negative control and the retrotransposon TA2 as the positive control.
(146 KB PDF)

Figure S4 Agreement between McrBC and meCIP-Based Methylation Microarrays McrBC and two meCIP-based methylation detection profiles are compared along 125 kB of euchromatin 9 Mb from the nuclear organizing region. Open reading frames from genes (yellow) and retrotransposons (green) are indicated, along with repeats predicted by RepBase and TandemRepeatFinder. Small RNA matches from massively parallel signature-sequencing (MPSS) data are indicated by arrowheads. Tiles that detect significant differences in copy number are highlighted in purple (CGH), while tiles that detect significant DNA methylation by means of McrBC-based detection are highlighted in red for the two land races (5 mC, Col and 5 mC, Ler). For comparison, the posterior probability of methylation at 35-bp microarray probes as determined by mCIP-based detection [27] and significant uncorrected log_2_ ratios of mCIP-enriched DNA to input DNA for 220-bp probes [28] is shown for WT Col-0. The three detection protocols show significant agreement, especially for more heavily methylated features such as repetitive elements.
(195 KB PDF)

Figure S5 Detection of Gene and TE Methylation by McrBC and meCIP Microarray Technologies Methylated transcription units annotated as Genes or TEs are compared on a per-open reading frame basis between the McrBC-based tiling array and two mCIP-based methods [27,28].
(17 KB PDF)

Protocol S1 Supplemental Methods, Analyses, and References Details of bioinformatic analyses, microarray design and printing, DNA extraction, microarray hybridizations, and statistical analysis are provided. In addition, a detailed comparison of McrBC and immunological methylation microarray results is presented.
(1.5 MB DOC)

Table S1 Genes Exhibiting Significant CNP in Col or Ler Genes on Chromosome 4 that have significant CNP between Col and Ler based on CGH are listed, including those identified by Borevitz et al [32]. Increase or decrease in Ler relative to Col is annotated as a plus or minus. Agreements between our data and the Borevitz CNPs are noted in the third column.
(529 KB DOC)

Table S2 Validation of Methylation Status in Col and Ler at 28 Loci and Comparison to Immunopurification-Based Approaches A total of 28 microarray feature were selected, their methylation status in Col and Ler assayed by McrBC digestion followed by PCR amplification, and the results compared to microarray-derived predictions (Materials and Methods). In addition, microarray data from two recent methylation profiling studies [27,28] were interpolated onto our arrays and compared to empirical results for these loci (see Protocol S1 for full discussion). In each case, + indicates methylation, − indicates lack thereof. Red cells highlight discrepancies relative to McrBC-PCR assay results.
(96 KB DOC)

Table S3 Summary of Methylation Inheritance Study Results for two genes with ecotype-specific methylation from genotyping and McrBC + PCR based methylation assays for two generations of progeny from reciprocal crosses between Col and Ler. Values in parentheses indicate number of progeny having methylation at each gene. Sample data for *At4g28850* are presented in Figure 4.
(30 KB DOC)

Table S4 Correlation between Ler CNPs and Expression Levels Expression levels for Affymetrix *ATH1* probe sets falling within tiles with putative deletions in Ler were compared in Col and Ler four-day seedlings using available microarray data (Materials and Methods). Because the microarray is based on the Col reference sequence, Col deletions cannot be detected (ND). The expected number of tiles in each class, assuming no correlation, are indicated in parentheses. Of the probe sets overlapping deletions, 13% were expressed only in Col. Furthermore, there were a greater than expected number of genes deleted in Ler that were not expressed at all in Col, suggesting that at least some of these may be pseudogenes.
(27 KB DOC)

Table S5 Methylation Polymorphism Versus Gene Expression Genes were classified by their methylation status in Col and Ler into “none,” where there was no detectable methylation in either ecotype, “both,” where at least one methylated tile was found in both ecotypes, “Col only” where methylation was only detectable in Col, and “Ler only” where methylation was only detectable in Ler. Expression of these classes of genes was examined in Col and Ler four-day seedlings [41]. The expected number of genes in each class, assuming no correlation, are indicated in parentheses. No significant differences in expression could be attributed to the presence of interecotype methylation polymorphisms.
(34 KB DOC)

Table S6 Unannotated Tiles Selected for use in Statistical Analysis of Methylation Microarray Data A total of 576 tiles with no gene, repeat, or TE annotation were selected at random as a basis for calculating the mean euchromatic methylation signal.
(50 KB DOC)

Table S7 Primer Sequences used in McrBC-PCR for Figures 1 and 3
Primers were used to amplify a fragment from McrBC or mock-digested samples to assay DNA cytosine methylation.
(34 KB DOC)

Table S8 Annotation-Based Summary Comparison of Our Microarray Results to Other Methylation Profiling Studies Methylation status of tiles on the Chromosome 4 tiling microarray was inferred from recently published immunologically derived data and is compared to the present McrBC-based results. These comparisons are stratified by tile annotation class.
(31 KB DOC)

Table S9 Annotation of Microarray Features Profiled in Natural Variation Study Open reading frames corresponding to the microarray features profiled for DNA cytosine methylation in 96 *Arabidopsis* natural variation accessions.
(54 KB DOC)

### Accession Numbers

The Gene Expression Omnibus (GEO) (http://www.ncbi.nlm.nih.gov/geo) accession number discussed in this paper is GSE7580.

The *Arabidopsis* Information Resource (TAIR) (http://www.arabidopsis.org) accession number for Col and Ler expression profiles is 1008803961.

## Abbreviations

CGH: comparative genome hybridization
CNP: copy number polymorphism
Col: Columbia
Ler: Landsberg *erecta*
siRNA: small interfering RNA
TE: transposable element
WT: wild type

## References

[pbio-0050174-b001] Bender J (2004). DNA methylation and epigenetics. Annu Rev Plant Biol.

[pbio-0050174-b002] Chan SW, Henderson IR, Jacobsen SE (2005). Gardening the genome: DNA methylation in Arabidopsis thaliana. Nat Rev Genet.

[pbio-0050174-b003] Klose RJ, Bird AP (2006). Genomic DNA methylation: The mark and its mediators. Trends Biochem Sci.

[pbio-0050174-b004] Terranova R, Agherbi H, Boned A, Meresse S, Djabali M (2006). Histone and DNA methylation defects at Hox genes in mice expressing a SET domain-truncated form of Mll. Proc Natl Acad Sci U S A.

[pbio-0050174-b005] Weber M, Davies JJ, Wittig D, Oakeley EJ, Haase M (2005). Chromosome-wide and promoter-specific analyses identify sites of differential DNA methylation in normal and transformed human cells. Nat Genet.

[pbio-0050174-b006] Gehring M, Choi Y, Fischer RL (2004). Imprinting and seed development. Plant Cell.

[pbio-0050174-b007] Sung S, Amasino RM (2005). Remembering winter: Toward a molecular understanding of vernalization. Annu Rev Plant Biol.

[pbio-0050174-b008] Alleman M, Sidorenko L, McGinnis K, Seshadri V, Dorweiler JE (2006). An RNA-dependent RNA polymerase is required for paramutation in maize. Nature.

[pbio-0050174-b009] Woodhouse MR, Freeling M, Lisch D (2006). Initiation, establishment, and maintenance of heritable MuDR transposon silencing in maize are mediated by distinct factors. PLoS Biol.

[pbio-0050174-b010] Silva AJ, White R (1988). Inheritance of allelic blueprints for methylation patterns. Cell.

[pbio-0050174-b011] Messeguer R, Ganal MW, Steffens JC, Tanksley SD (1991). Characterization of the level, target sites and inheritance of cytosine methylation in tomato nuclear DNA. Plant Mol Biol.

[pbio-0050174-b012] Lippman Z, May B, Yordan C, Singer T, Martienssen R (2003). Distinct mechanisms determine transposon inheritance and methylation via small interfering RNA and histone modification. PLoS Biol.

[pbio-0050174-b013] Morgante M, Brunner S, Pea G, Fengler K, Zuccolo A (2005). Gene duplication and exon shuffling by helitron-like transposons generate intraspecies diversity in maize. Nat Genet.

[pbio-0050174-b014] Jiang N, Bao Z, Zhang X, Eddy SR, Wessler SR (2004). Pack-MULE transposable elements mediate gene evolution in plants. Nature.

[pbio-0050174-b015] Rabinowicz PD, Palmer LE, May BP, Hemann MT, Lowe SW (2003). Genes and transposons are differentially methylated in plants, but not in mammals. Genome Res.

[pbio-0050174-b016] Peaston AE, Whitelaw E (2006). Epigenetics and phenotypic variation in mammals. Mamm Genome.

[pbio-0050174-b017] Lippman Z, Gendrel AV, Black M, Vaughn MW, Dedhia N (2004). Role of transposable elements in heterochromatin and epigenetic control. Nature.

[pbio-0050174-b018] Saze H, Scheid OM, Paszkowski J (2003). Maintenance of CpG methylation is essential for epigenetic inheritance during plant gametogenesis. Nat Genet.

[pbio-0050174-b019] Xiao W, Custard KD, Brown RC, Lemmon BE, Harada JJ (2006). DNA methylation is critical for *Arabidopsis* embryogenesis and seed viability. Plant Cell.

[pbio-0050174-b020] Singer T, Yordan C, Martienssen RA (2001). Robertson's Mutator transposons in A. thaliana are regulated by the chromatin-remodeling gene Decrease in DNA Methylation (DDM1). Genes Dev.

[pbio-0050174-b021] Miura A, Yonebayashi S, Watanabe K, Toyama T, Shimada H (2001). Mobilization of transposons by a mutation abolishing full DNA methylation in *Arabidopsis*. Nature.

[pbio-0050174-b022] Vongs A, Kakutani T, Martienssen RA, Richards EJ (1993). Arabidopsis thaliana DNA methylation mutants. Science.

[pbio-0050174-b023] Kakutani T (2002). Epi-alleles in plants: Inheritance of epigenetic information over generations. Plant Cell Physiol.

[pbio-0050174-b024] Henderson IR, Zhang X, Lu C, Johnson L, Meyers BC (2006). Dissecting Arabidopsis thaliana DICER function in small RNA processing, gene silencing and DNA methylation patterning. Nat Genet.

[pbio-0050174-b025] Kato M, Miura A, Bender J, Jacobsen SE, Kakutani T (2003). Role of CG and non-CG methylation in immobilization of transposons in *Arabidopsis*. Curr Biol.

[pbio-0050174-b026] Tran RK, Henikoff JG, Zilberman D, Ditt RF, Jacobsen SE (2005). DNA methylation profiling identifies CG methylation clusters in *Arabidopsis* genes. Curr Biol.

[pbio-0050174-b027] Zhang X, Yazaki J, Sundaresan A, Cokus S, Chan SW (2006). Genome-wide high-resolution mapping and functional analysis of DNA methylation in *Arabidopsis*. Cell.

[pbio-0050174-b028] Zilberman D, Gehring M, Tran RK, Ballinger T, Henikoff S (2007). Genome-wide analysis of Arabidopsis thaliana DNA methylation uncovers an interdependence between methylation and transcription. Nat Genet.

[pbio-0050174-b029] Bender J (2004). DNA methylation of the endogenous PAI genes in *Arabidopsis*. Cold Spring Harb Symp Quant Biol.

[pbio-0050174-b030] Lippman Z, Gendrel AV, Colot V, Martienssen R (2005). Profiling DNA methylation patterns using genomic tiling microarrays. Nat Methods.

[pbio-0050174-b031] Martienssen RA, Doerge RW, Colot V (2005). Epigenomic mapping in *Arabidopsis* using tiling microarrays. Chromosome Res.

[pbio-0050174-b032] Borevitz JO, Liang D, Plouffe D, Chang HS, Zhu T (2003). Large-scale identification of single-feature polymorphisms in complex genomes. Genome Res.

[pbio-0050174-b033] Arabidopsis Genome Initiative (2000). Analysis of the genome sequence of the flowering plant Arabidopsis thaliana. Nature.

[pbio-0050174-b034] Madlung A, Tyagi AP, Watson B, Jiang H, Kagochi T (2005). Genomic changes in synthetic *Arabidopsis* polyploids. Plant J.

[pbio-0050174-b035] Frank MJ, Preuss D, Mack A, Kuhlmann TC, Crawford NM (1998). The *Arabidopsis* transposable element Tag1 is widely distributed among *Arabidopsis* ecotypes. Mol Gen Genet.

[pbio-0050174-b036] Sebat J, Lakshmi B, Troge J, Alexander J, Young J (2004). Large-scale copy number polymorphism in the human genome. Science.

[pbio-0050174-b037] Gendrel AV, Lippman Z, Martienssen R, Colot V (2005). Profiling histone modification patterns in plants using genomic tiling microarrays. Nat Methods.

[pbio-0050174-b038] Bao N, Lye KW, Barton MK (2004). MicroRNA binding sites in *Arabidopsis* class III HD-ZIP mRNAs are required for methylation of the template chromosome. Dev Cell.

[pbio-0050174-b039] Chan SW, Henderson IR, Zhang X, Shah G, Chien JS (2006). RNAi, DRD1, and histone methylation actively target developmentally important non-CG DNA methylation in *Arabidopsis*. PLoS Genet.

[pbio-0050174-b040] Lu C, Tej SS, Luo S, Haudenschild CD, Meyers BC (2005). Elucidation of the small RNA component of the transcriptome. Science.

[pbio-0050174-b041] Lempe J, Balasubramanian S, Sureshkumar S, Singh A, Schmid M (2005). Diversity of flowering responses in wild Arabidopsis thaliana strains. PLoS Genet.

[pbio-0050174-b042] Aranzana MJ, Kim S, Zhao K, Bakker E, Horton M (2005). Genome-wide association mapping in *Arabidopsis* identifies previously known flowering time and pathogen resistance genes. PLoS Genet.

[pbio-0050174-b043] Nordborg M, Hu TT, Ishino Y, Jhaveri J, Toomajian C (2005). The pattern of polymorphism in Arabidopsis thaliana. PLoS Biol.

[pbio-0050174-b044] Soppe WJ, Jacobsen SE, Alonso-Blanco C, Jackson JP, Kakutani T (2000). The late flowering phenotype of fwa mutants is caused by gain-of-function epigenetic alleles of a homeodomain gene. Mol Cell.

[pbio-0050174-b045] Kinoshita T, Miura A, Choi Y, Kinoshita Y, Cao X (2004). One-way control of FWA imprinting in *Arabidopsis* endosperm by DNA methylation. Science.

[pbio-0050174-b046] Kinoshita Y, Saze H, Kinoshita T, Miura A, Soppe WJ (2007). Control of FWA gene silencing in Arabidopsis thaliana by SINE-related direct repeats. Plant J.

[pbio-0050174-b047] Genereux DP, Miner BE, Bergstrom CT, Laird CD (2005). A population-epigenetic model to infer site-specific methylation rates from double-stranded DNA methylation patterns. Proc Natl Acad Sci U S A.

[pbio-0050174-b048] Bestor TH, Ingram VM (1983). Two DNA methyltransferases from murine erythroleukemia cells: Purification, sequence specificity, and mode of interaction with DNA. Proc Natl Acad Sci U S A.

[pbio-0050174-b049] Cubas P, Vincent C, Coen E (1999). An epigenetic mutation responsible for natural variation in floral symmetry. Nature.

[pbio-0050174-b050] Jacobsen SE, Meyerowitz EM (1997). Hypermethylated SUPERMAN epigenetic alleles in *Arabidopsis*. Science.

[pbio-0050174-b051] Richards EJ (2006). Inherited epigenetic variation–revisiting soft inheritance. Nat Rev Genet.

[pbio-0050174-b052] Chan SW, Zilberman D, Xie Z, Johansen LK, Carrington JC (2004). RNA silencing genes control de novo DNA methylation. Science.

[pbio-0050174-b053] Lippman Z, Martienssen R (2004). The role of RNA interference in heterochromatic silencing. Nature.

[pbio-0050174-b054] Turck F, Roudier F, Farrona S, Martin-Magniette M, Guillaume E (2007). *Arabidopsis* TFL2/LHP1 specifically associates with genes marked by trimethylation of histone H3 lysine 27. PLOS Genet.

[pbio-0050174-b055] Kalisz S, Purugganan MD (2004). Epialleles via DNA methylation: Consequences for plant evolution. Trends Ecol Evol.

[pbio-0050174-b056] Gehring M, Huh JH, Hsieh TF, Penterman J, Choi Y (2006). DEMETER DNA glycosylase establishes MEDEA polycomb gene self-imprinting by allele-specific demethylation. Cell.

[pbio-0050174-b057] Cervera MT, Ruiz-Garcia L, Martinez-Zapater JM (2002). Analysis of DNA methylation in Arabidopsis thaliana based on methylation-sensitive AFLP markers. Mol Genet Genomics.

[pbio-0050174-b058] Craig BA, Black MA, Doerge RW (2003). Gene expression data: The technology and statistical analysis. Journal of Agricultural Biological and Environmental Statistics.

[pbio-0050174-b059] Smyth GK (2004). Linear models and empirical Bayes methods for assessing differential expression in microarray experiments. Stat Appl Genet Mol Biol.

[pbio-0050174-b060] Jurka J, Kapitonov VV, Pavlicek A, Klonowski P, Kohany O (2005). Repbase Update, a database of eukaryotic repetitive elements. Cytogenet Genome Res.

[pbio-0050174-b061] Noel L, Moores TL, van Der Biezen EA, Parniske M, Daniels MJ (1999). Pronounced intraspecific haplotype divergence at the RPP5 complex disease resistance locus of *Arabidopsis*. Plant Cell.

