# Supplementary material for: Epigenetic Natural Variation in Arabidopsis thaliana
Source: PLoS Biol. 2007 Jun 19;5(7):e174. doi: 10.1371/journal.pbio.0050174 (PMC1892575; doi:10.1371/journal.pbio.0050174)
Supplement: Supplementary file 2 [file pbio.0050174.sg002.pdf]

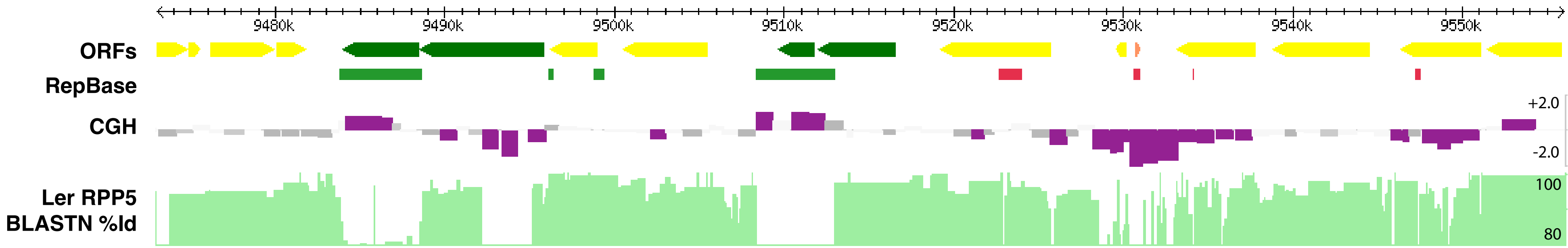

9480k
9490k
9500k
9510k
9520k
9530k
9540k
9550k
ORFs
RepBase
CGH
+2.0
-2.0
Ler RPP5
BLASTN %Id
100
80

Figure S2
