# Supplementary material for: Epigenetic Natural Variation in Arabidopsis thaliana
Source: PLoS Biol. 2007 Jun 19;5(7):e174. doi: 10.1371/journal.pbio.0050174 (PMC1892575; doi:10.1371/journal.pbio.0050174)
Supplement: Supplementary file 3 [file pbio.0050174.sg003.pdf]

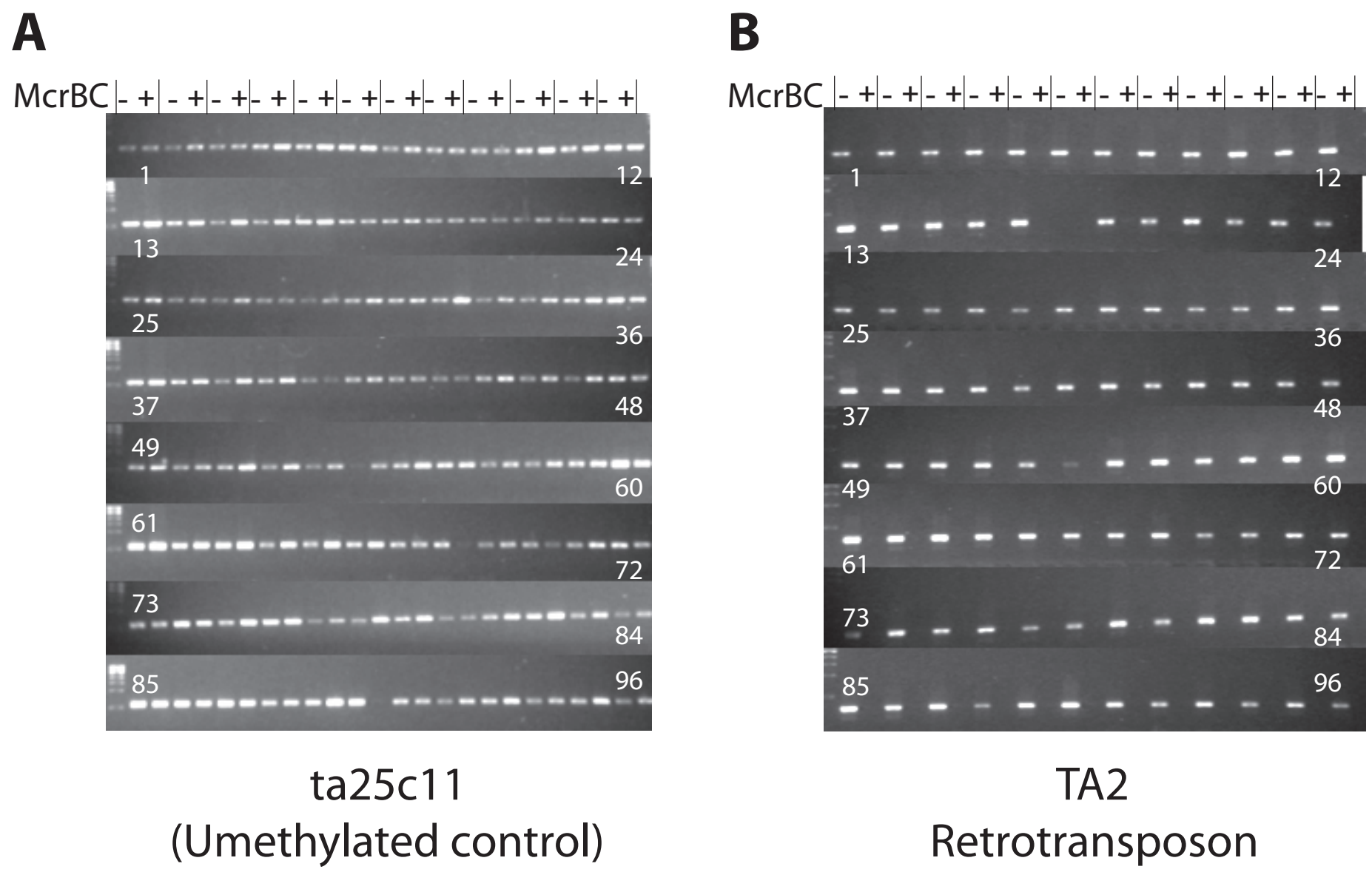
A
McrBC
- + - + - + - + - + - + - + - + - + - + - + - +
1
12
13
24
25
36
37
48
49
60
61
72
73
84
85
96
ta25c11
(Umethylated control)
B
McrBC
- + - + - + - + - + - + - + - + - + - + - + - +
1
12
13
24
25
36
37
48
49
60
61
72
73
84
85
96
TA2
Retrotransposon

Figure S3
