# Supplementary material for: Epigenetic Natural Variation in Arabidopsis thaliana
Source: PLoS Biol. 2007 Jun 19;5(7):e174. doi: 10.1371/journal.pbio.0050174 (PMC1892575; doi:10.1371/journal.pbio.0050174)
Supplement: Supplementary file 4 [file pbio.0050174.sg004.pdf]

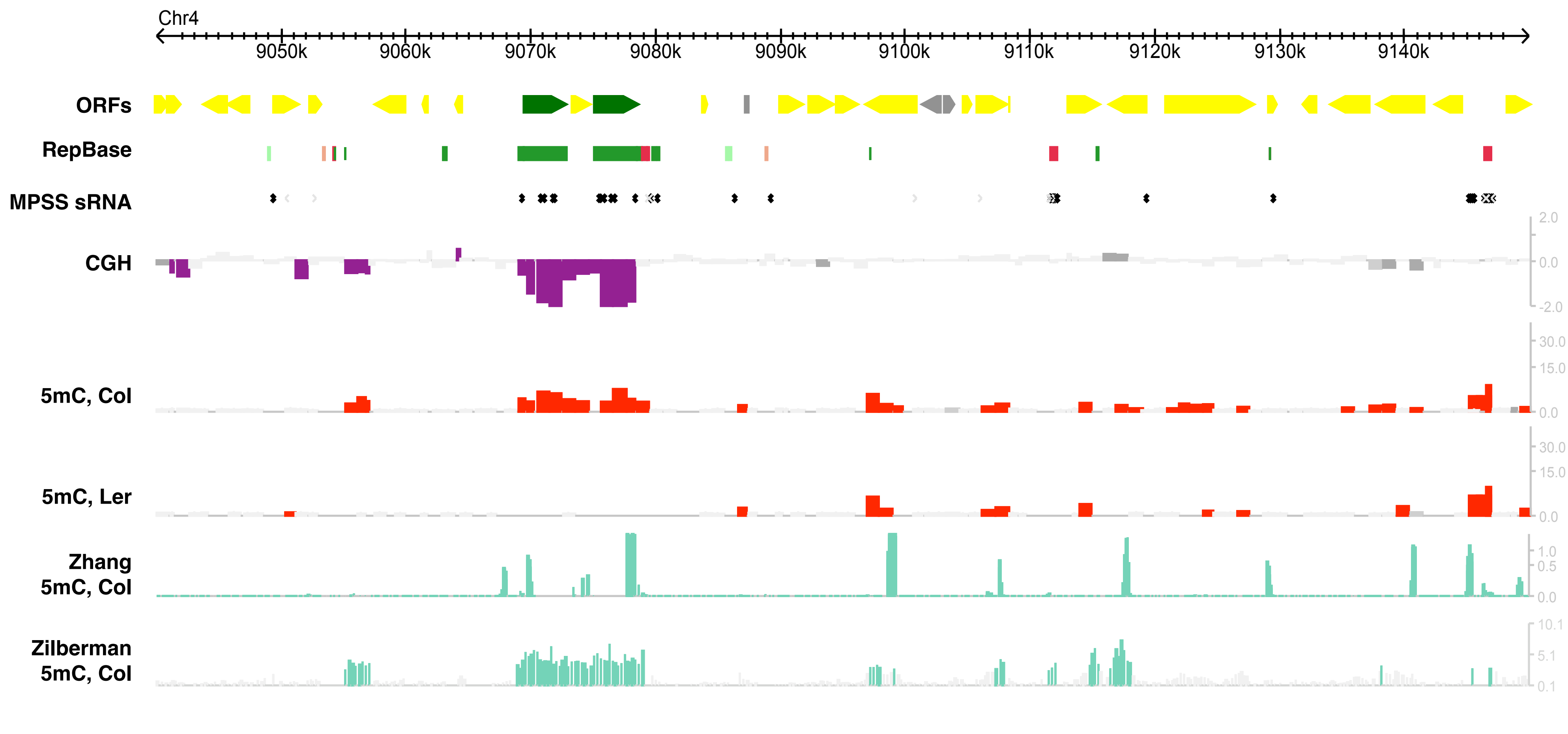
Chr4
9050k
9060k
9070k
9080k
9090k
9100k
9110k
9120k
9130k
9140k
ORFs
RepBase
MPSS sRNA
CGH
2.0
0.0
-2.0
5mC, Col
30.0
15.0
0.0
5mC, Ler
30.0
15.0
0.0
Zhang
5mC, Col
1.0
0.5
0.0
Zilberman
5mC, Col
10.1
5.1
0.1

Figure S4
