# Supplementary material for: Epigenetic Natural Variation in Arabidopsis thaliana
Source: PLoS Biol. 2007 Jun 19;5(7):e174. doi: 10.1371/journal.pbio.0050174 (PMC1892575; doi:10.1371/journal.pbio.0050174)
Supplement: Supplementary file 5 [file pbio.0050174.sg005.pdf]

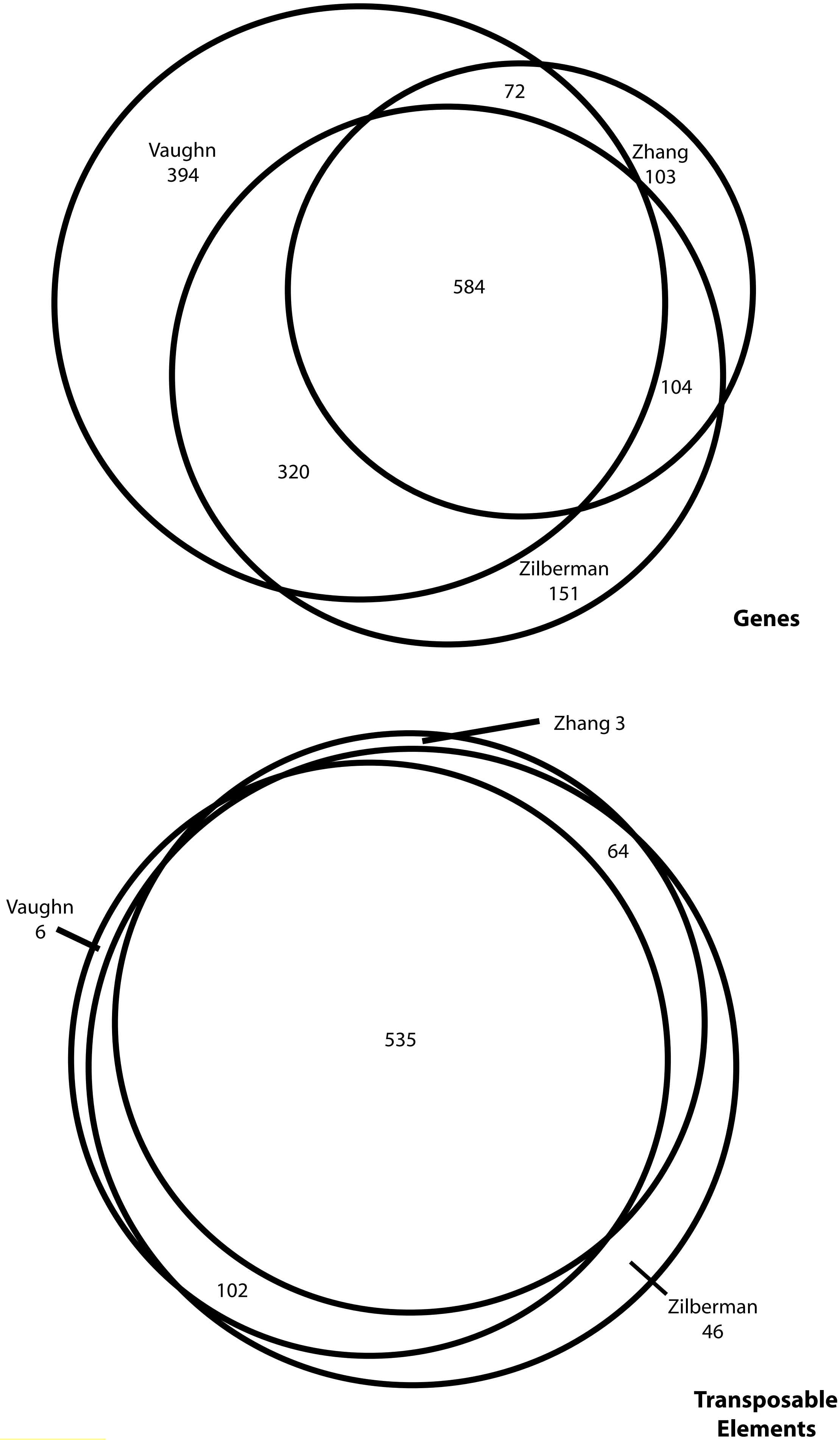
72
Vaughn
394
Zhang
103
584
104
320
Zilberman
151
Genes
Zhang 3
64
Vaughn
6
535
102
Zilberman
46
Transposable
Elements

Figure S5
