# Supplementary material for: Epigenetic Natural Variation in Arabidopsis thaliana
Source: PLoS Biol. 2007 Jun 19;5(7):e174. doi: 10.1371/journal.pbio.0050174 (PMC1892575; doi:10.1371/journal.pbio.0050174)
Supplement: Supplementary file 6 [file pbio.0050174.sd001.doc]

# Protocol S1

## Methods

**Overview and data submission.**

Methods used in the design, hybridisation and analysis of the microarrays are given below. The microarray platform (GPL5116) and associated experimental series (GSE7580) have been submitted to the NCBI Gene Expression Omnibus (http://www.ncbi.nlm.nih.gov/geo/).

**Bioinformatics and website**

The Arabidopsis genome annotation used in this analysis was based on the XML pseudomolecules from the TAIR 2006 reference release (http://www.arabidopsis.org/). Microarray tile positions were mapped from TIGR4 (used in design of array) using *in silico* PCR. Additional physical features were added to the annotation by stringent BLASTN. Repeat and transposon sequences were annotated by CENSOR analysis [1] with version 10.4 of RepBase [2] using normal stringency. Methylation and CGH profiles were loaded into a MySQL relational database implementing the Bio::DG::GFF schema, thus facilitating intersecting positional, quantitative, and class-based queries and computations. A browser-based interface to the data is available at our website, <http://chromatin.cshl.edu/epivariation/>. This website allows user-defined displays of our microarray data type aligned with genome sequence annotations and various other data types. Queries by physical location or gene identifier, as well as more general queries guided by a map of chromosome 4, can be used to simultaneously access data from multiple experiments and annotation sources

**Microarray design and printing.**

The entire TIGR4 Arabidopsis thaliana ec. Col chromosome 4 assembly was used to design a genomic tiling microarray. This ~18.6 Mb sequence represented on the microarray starts downstream of the nucleolar organizing region (NOR) comprising the 5’ terminus of the chromosome. Notably, it contains several megabases of pericentromeric heterochromatin. In the microarray design, annotation was intentionally ignored, thus ensuring that repeated and unique sequences were equally represented. PCR primer pairs were selected using the Primer3 software. Probe selection was first accomplished by BLASTN analysis of sequential 100-bp windows of sequence along chromosome 4 against the whole genome to discriminate between unique and repeated DNA. Whenever a window gave at least one hit with 85% identity elsewhere in the genome, that window was marked as repeated, or unique otherwise. This information was then used to define "repeated" and "unique" segments of at least 300bp along the chromosome, as follows. Repeat segments were composed of successive repeat windows, possibly interrupted by series of contiguous unique windows spanning less than 300bp in each case. Conversely, unique segments were composed of successive unique windows, never interrupted by more than 300bp of contiguous repeat windows. Series of alternate unique and repeated windows were treated as repeat segments. Within each repeated or unique segment, PCR primer pairs were then selected using the Primer3 software and a routine that ensured maximum coverage of the segment and limited overlap between successive probes. Briefly, primer pairs were designed from both ends of the segment, considering sequential 1.2 kb windows, amplicon sizes ranging from 850 bp to 1.2 kb (optimum 1 kb) and primers (19-23 nt each, optimum 21 nt, 40-60% GC) with similar melting temperatures in the 57°C­63°C range (optimum 60°C). Each new 1.2 kb window was positioned 50 bp within the end of the preceding amplicon. Successive amplicons could therefore overlap by up to 50 bp or be separated by up to 250 bp. When no primer pair was found in a given window, window size was increased to 1.4 kb, and if still unsuccessful, amplicons were finally designed using the forward and reverse primers of the upstream and downstream amplicons, respectively. When the last (central) window of a segment was less than 1.2kb but more than 300bp, primer pair selection was carried out by considering the actual size of that window plus 50bp on either side. Using this approach, 21,405 amplicons were designed with an average size of ~950bp. An additional 356 amplicons were designed that cover 36 genes of interest and neighboring sequences located on the other 4 chromosomes. Most amplicons were produced using BAC clones as templates, and all amplicons were verified by gel electropheresis, as previously described [3].

PCR was performed in 100 ul volume using 100 pmole of each primer, and either BAC (20-40 pg) or genomic (100ng) DNA. Reactions were systematically analyzed by agarose gel electrophoresis. Approximately 5% of PCRs failed to give any product or gave multiple products or products of the wrong size. PCR products (2-8ug) were purified using Millipore Multiscreen FB Plates (MAFBN0B50), eluted in 60ul of 50% DMSO, and dispensed in 1536-well plates. Microarray slides (UltraGAPS, Corning) were printed using a MicroGrid arrayer (Genomic Solutions).

**DNA extraction and labeling**

CGH and DNA methylation experiments were performed with genomic DNA from 14-days old seedlings from a pool of about a hundred plants. DNA extraction was performed in duplicate using the Qiagen Plant DNA Easy kit. Previous estimates of intra vs inter-ecotype methylation variability estimated the intra-ecotype variability an order of magnitude lower than the inter-ecotype variation [4], therefore pooled DNA was used in all analyses.

**Comparative Genome Hybridization (CGH)**

Ten micrograms of genomic DNA was sheared to an average size of 1-3kb using nebulizers, as described in detail in [5]. Three micrograms of the DNA sample were labeled with Cy3 or Cy5 dyes by random priming [5] and purified over Qiagen PCR cleanup columns according to the supplier’s protocol. Equal amounts of Col DNA labeled with Cy3 or Cy5 and Ler DNA labeled with Cy5 or Cy3, respectively, were mixed for hybridization to a single array in 4X SSC and 0.2% SDS. Arrays were hybridized at 65C for 16-18 hrs, followed by 4 washes (1X SSC 0.2% SDS for 5 minutes at 42C, followed by 1X SSC at room temperature for 5 minutes and two washes in 0.1X SSC at room temperature for 2 minutes). Arrays were immediately scanned using Axon 4000 array scanner and the scans were quantified with GenePix v4.0. A single complete set of hybridizations consisted of a dye-swap (Col-Cy3 + Ler-Cy5 vs Col-Cy5 + Ler-Cy3). Three technically replicated dye swaps were performed (see Discussion).

## Cytosine methylation analysis

Genomic DNA sheared the same way as in the CGH experiment was used for enzymatic digestion of DNA methylated at sites recognized by mcrBC (5’ (G/A)^m^C(N)_40-3000_(G/A)^m^ 3’). After digestion, the sample, as well as the same amount of mock-treated, sheared DNA, was run briefly, side-by-side on a small 1% agarose gel. DNA fractions larger than 800-1000bp were cut from the gel and DNA purified using Qiagen Qiaex II resin. In this way, the mcrBC-treated sample represents enrichment of un-methylated portion of the genome (this approach to detecting DNA methylation on microarrays has recently been shown to be better adept at detecting quantitative differences in DNA methylation than methods that preferentially enrich the methylated portion [6]). Upon digestion, both the sheared and the mcrBC-treated DNA were randomly amplified using procedure described in [5]. Fluorescent DNA labeling and microarray hybridization was performed identically to CGH experiments. In order to detect methylation in the Col and Ler ecotypes, the sheared (non-mcrBC treated) and the digested (mcrBC-treated) DNA from the same ecotype were hybridized on one array, with a dye-swap for each pair of samples. In this way, the potential difference in fluorescence signals between Cy3 and Cy5 channels is not confounded by the copy-number differences between ecotypes. Three replicates of each dye-swap set were performed for both Col and Ler ecotypes to account for technical variation.

**Statistical analysis of microarray data**

The methylation intensity for each spot is computed by subtracting the background median intensity from the corresponding foreground median intensity for both r signals. If the foreground intensity is less than the background intensity, the methylation level is set as 1 (log-transformed intensity 0). To stabilize the variance, log_2_ transformation is applied to the background corrected intensities. MA plots [7] revealed a general trend across all the slides for the Cy5 to yield higher measurements than Cy3 (Figs. 1 and 2). To correct this, a robust local regression (loess) [8] was used to remove the unbalanced dye effect using all features on the array (the green lines in MA plots, Figs.1 and 2). After the normalization, the log-ratio, M, has a mean of approximately zero.

**Figure 1*. MA plots for Col-0 (methylation) triplicated dye-swaps***

**Figure 2. *MA plots for Ler (methylation) triplicated dye-swaps***

To illustrate the hybridization of Ler to the reference Col genome, the magnitude of feature intensity for Ler is compared to Col. It is anticipated that if Ler hybridizes similarly to Col on the microarray, the feature intensities between Ler and Col are at comparable levels. As shown in Figure 3, most features hybridize more strongly to Col than Ler targets. Figure * illustrates the MA plots for each of the six slides. If a feature (probe) shows no difference in hybridization intensity between Col and Ler, the log-ratio, M, is close to 0. If a feature hybridizes more (or less) stringently to the sample labeled with Cy5 dye, then it is above (or below) the horizontal axis. As illustrated there is a trend across all six slides indicating that features (probes) hybridize more strongly to Col than in Ler, as expected given that the tiling array is constructed based on Col genome sequence.

**Figure 3. *MA plots for each of the six slides Ler is hybridized to the reference Col genome. The dotted blue line is the loess smoothing line.***

Both the CGH and methylation genomic tiling microarray data were analyzed separately using a linear model analysis of variance (ANOVA) (for details see [9]). Specifically, a linear model of the form

ln(Y_ijkmr_)= µ + A_i_ +D_i_ +T_k_+G_m_+AG_im_+DG_jm_+TG_km_ +ε_ijkmr_

was employed to partition the sources of variation. The natural logarithm transformed, raw, background corrected data are denoted as ln(Y_ijkmr_). The overall mean effect is µ, and A, D, T, and G represent the array, dye, treatment, and gene (or feature) main effects, respectively. The interactions of the main effects are AG, DG, TG, and represent array by gene, dye by gene, and treatment by gene, respectively. The random error ε_ijkmr_ is assumed to be normally distributed, with mean zero and constant variance. Once the sources of technical variation (e.g., global and feature-specific array and dye effects) and experimental variation (e.g., treatment and treatment by feature interaction) are estimated, differential fluorescence in the CGH experiment was tested using hypotheses that acknowledge both the average treatment effect and the treatment by feature interaction [10]:

H_0_:T_k_+TG_km_=T_k’_+TG_k’m_

H_a_: T_k_+TG_km_ ≠T_k_’+TG_k’m_.

Statistically significant differences were determined by evaluating signals at each tile using a two-sided t-test. For the CGH experiments each feature on the array is compared to the entire tiling array. However, when testing for differential methylation it was necessary to use control features that enabled the detection of subtle signals coming from euchromatic methylation, as well as heterochromatin. To accomplish this, 576 randomly selected tiles across chromosome 4 regions with no gene or repeat annotation were used as controls (Supplementary Table 6). These controls supplied an average unmethylated value against which other features on chromosome were tested using a one-sided t-test. For both the CGH and methylation experiments the multiple testing issues that arise when testing 21,815 tiles across chromosome 4 the false discovery rate (FDR) were controlled at the 5% level using a Benjamini and Hochberg correction.

Summary information is presented for the analysis of the Col and Ler methylation dye-swap experiments . An ANOVA table is presented for the Col and Ler dye-swap experiments and provides estimates for the sources of variation that are acknowledged in the linear model. As shown by scatter plots of the residuals (and standard deviation against mean feature intensity) from the fitted linear model indicates lower signal strengths are subject to increased random variation. Figure 4 illustrates the mean log-fold methylation changes (mock/mcrBC treated) for each feature that resulted from fitting the previously mentioned linear model. Statistically significant and multiple testing-corrected differentially methylated features are highlighted in both pink (FDR) and blue (FDR-Holm intersection). Results from the ANOVA using a per-gene variance assumption are shown along side comparative results from an ANOVA assuming a common variance. Features are plotted in linear order along chromosome 4, except for those above 18,000, which are from more recently sequenced inner pericentromeric repeats. As illustrated, heterochromatic features have much higher mean log-fold change than euchromatic features. After comparing to the common variance assumption all further analyses, results and discussion are based on an ANOVA using a per-gene variance with an FDR of 0.05.

**Figure 4. *Summary information for testing methylation differences in Col-0 using a triplicated dye-swap experimental design.***

**Figure 5. *Summary information for testing methylation differences in Ler using a triplicated dye-swap experimental design.***

Similarly, summary information is presented for the analysis of the Col and Ler CGH duplicated dye-swap experiments . An ANOVA table is presented and provides estimates for the sources of variation that are acknowledged in the linear model. As shown by scatter plots of the residuals (and standard deviation against mean feature intensity) from the fitted linear model indicates lower signal strengths are subject to increased random variation. Figure 5 illustrates the mean log-fold genomic changes for each feature that resulted from fitting the previously mentioned linear model. Statistically significant multiple testing corrected differentially features highlighted in both pink (FDR) and blue (FDR-Holm intersection). Results from the ANOVA using a per-gene variance assumption are shown along side comparative results from an ANOVA assuming a common variance. Features are plotted in linear order along chromosome 4, except for those above 18,000, which are from more recently sequenced inner pericentromeric repeats. As illustrated, most significant changes are “insertions” in Columbia relative to Ler, which are represented as a log fold-change below 1.0. This is because the tiling array is based on the sequence of Col and therefore incapable of detecting insertions in Ler of DNA sequences absent from Col. For simplicity, these are referred to in the text as deletions from Ler. After comparing to the common variance assumption all further analyses, results and discussion are based on an ANOVA using a per-gene variance with an FDR of 0.05.

**Figure 6. *Summary information for testing comparative genome hybridization between Ler and the reference genome Col-0 using a triplicated dye-swap experimental design.***

## Analysis

**Comparison of McrBC- and immunopurification-based methylation profiling results**

Recently, Zhang et al. [11] monitored DNA methylation profiles in Arabidopsis by probing an Affymetrix tiling microarray comprising 25nt oligos selected every 35bp from the genome sequence with DNA enriched using anti-5mC antibodies. On these chips, repeated oligonucleotides were represented only once resulting in approximately 75% of repetitive regions of the genome being discarded. Zilberman et al. [12] used a similar immunoenrichment protocol to probe microarrays with somewhat larger probe lengths (~55nt) spaced 220 bases apart. In general, our 1kb tiling arrays had one-half to one-third the resolution of these arrays (see below) and could distinguish between most repetitive tiles as well as detect features with lower degrees of methylation. Both Zhang et al. and Zilberman et al. used the mCIP method to which utilizes anti-5mC antibodies in a chromatin IP, while we used a restriction enzyme-based method. Nonetheless, the results for Columbia chromosome 4 are in good agreement with those presented here. For example, in all three studies, 19-33% of genes were found to have detectable methylation, usually in the central region of the gene, while 70-80% of transposons had dense methylation throughout the TE (Supplemental Figure 4 and Supplemental Table 8). These results are in close agreement with our previously published profiles of the short arm of chromosome 4 in which 29 out of 106 genes in this largely heterochromatic region were found to have low levels of methylation, but virtually all TEs had high levels.

Our results are also in close agreement concerning the impact of methylation on gene expression. Zhang et al. found that less than 2% of all genes were upregulated in the DNA methyltransferase mutant met1-3, compared with 25% of all genes that lost methylation in a mutant background, indicating that methylation had almost no effect on gene expression. Similarly, we found only 2% of genes that were differentially methylated between Landsberg and Columbia were also differentially expressed, and we previously reported that only 3% of genes on the short arm of chromosome 4 were upregulated in mutants which lose methylation [3]. In most of those cases, TEs or fragments thereof were found within the annotated transcription unit, and were likely responsible for upregulation. Similarly, the 319 genes upregulated in met1 mutants detected by Zhang et al. include 267 that have significant homology to TEs and may be regulated in a similar way. Of these 319 genes, 202 are methylated in WT, suggesting that their regulation would proceed more like TEs than genes.

In a similar vein, Zilberman et al. showed that genes without methylation, on average, exhibited an upregulation of 14% relative to wild-type in the methyltransferase mutant met1-6. Open reading frames with methylation in the wild-type exhibited an additional 14% increase in mean expression in met1-6. Relative to commonly accepted fold-changes in the microarray literature, these increases may be of borderline significance. The general increase in transcription found in met1-6 may have been incurred by failure to sufficiently account for sample and array effects, leaving only the small difference between methylated and unmethylated genes to be accounted for. Although Zilberman et al. were careful to eliminate from consideration genes corresponding to known transposable elements, not all such elements can be easily identified. For example, until April of 2006, the multicopy retroelement *Sahdu* [13] was not present in RepBase. Furthermore, some transposable element ORFs are sufficiently sequence-degenerate from their model sequence that only sensitive TBLASTX or peptide sequence clustering will reveal their identities. Finally, there are a number of genes that, while technically not TE genes by homology, must be considered as such for the purposes of this analysis. These genes are passengers on Helitron and Vandal class I transposons. In all three cases outlined here, open reading frames would be expected to be methylated and consequently overexpressed in *met1-6*. Consequently, the presence of only a handful of missed TEs or repeat-containing genes could account for a 14% expression difference.

In addition to questions of genomic annotation, technical differences lead to some variations in the findings derived from the three approaches. One major difference was in the effective resolution of the microarray analyses. Both Zhang et al. and Zilberman et al. used microarrays with smaller, more closely spaced probes than we did. In theory, this should have provided 28- and 5-fold increases, respectively, in array resolution relative to our array based on 1 kB PCR product probes. However, in both cases, the effective resolution was a function of the shear size of the immunopurified chromatin. To examine this, we determined the size distribution of significant methylation clusters based on these two similar approaches. For Zhang et al., who sonicated to an average size of 350 bases, the median cluster size was 348 nt, and for Zilberman et al. who sonicated to between 0.5 and 1.5 kB, the median cluster size was 660 nt. For our microarrays, the median cluster size was 1 kilobase. Thus, the 35 and 220 base-pair resolution arrays provide 2.8- and 1.5-fold increases, respectively, in resolution. Interestingly, the size distribution for the Zilberman et al. arrays was heavily skewed towards single-probe clusters while the Zhang array data exhibited a peak at 348 nt, suggesting that the Zilberman et al. data may contain among its collection of significant features additional noise relative to data from the other two arrays.

Although all three approaches used DNA extracted from pools of plants, effectively canceling out inter-plant variation in favor of identifying methylation domains common to a given ecotype, the source material for methylation profiling also differed between the three systems. Thus, some portion of the observed differences in methylation may be due to the fact that age of plant material profiled ranged from 14 to 35 days old. The older plants used by Zhang et al. may have included some inflorescence tissue, further complicating comparison of their results to ours and that of Zilberman et al. No exhaustive analysis of stage and tissue-specific methylation has been published. There should be good agreement between samples collected at any given time point based the excellent correlation of methylation results between of our lab strain of Col-0 to the CS22625 Nordborg Col-0 accession.

Finally, the three microarray studies differed significantly in their analytical methods. As described in the Supplementary Methods, we used a linear model ANOVA to partition the sources of variation, leading to a normalized estimates of per-probe methylation intensity as well as a P-values for each tile reflecting the likelhood that its value represented a significant treatment effect. In contrast, the Affymetrix tiling array probes used by Zhang et al. have a wide range of melting temperatures and a correspondingly large range of signal intensities, resulting in quite a bit of noise among the tiles comprising a given segment of the genome. For this reason it was necessary to use a Hidden Markov Model to integrate the replicated values from several adjacent tiles with elevated signals in order to detect methylation via an arbitrary cut-off of 0.5 on the associated posterior probability. The resulting P values do not reflect the level of methylation, only the likelihood that it exists within a given region. Zilberman et al. used longer, GC-normalized oligonucleotide probes, which can theoretically yield very high quality data. However, those experiments suffer from a lack of replication and corresponding paucity of statisical power. Because wild-type and met1-6 methylation profiles are based upon single hybridizations, per-probe error rates cannot be measured, making it impossible to know which signals represent genuine enrichment relative to the control channel. The authors resort to determining an abtritrary significance threshold that is based on the distribution of log2(M) values on the array, an approach that is certainly prone to error, as suggested by the abundance of single-probe significant features in the aforementioned clustering analysis.

Despite the differences in array resolution, source tissue, and analytical methodologies, agreement between the three approaches was generally quite good (Supplemental Table 8). On average, Zhang et al. detected methylation in around 80% of the same tiles we did. When these features were stratified by annotation class, 89% of methylated TE tiles and 64% of gene tiles were detected. The Zilberman et al. data, when analyzed under the assumption that all probes with log2(M) values greater than 1.28 represent significant signal, finds methylation in half again as many of our array features as we do, yet in 10% fewer tiles derived from genes. Experimental and analytical protocols for both of these higher resolution arrays, in their current forms, select for large (>350 nt) or highly enriched (2.4-fold) instances of methylation, neither of which is entirely compatible with identifying short clusters of gene methylation [14]. Consistent with this observation, when microarray features were consolidated on a per-open reading frame basis for comparison, agreement was much stronger among the three arrays for TE genes, compared to genes where our array approach seemed to be slightly better at identifying the lower levels of methylation (Supplemental Figure 5). Finally, we validated the Col-0 methylation status for 22 of 28 microarray features (79%) by McrBC digestion and PCR. By way of comparison, Zhang et al. detected the appropriate methylation status in 18 cases, while Zilberman agreed with the McrBC-PCR results in 20 of 28 cases (Supplemental Table 2). Zhang detected potential methylation at 3 tiles that was below their P=0.5 cutoff, suggesting that short or low-density regions of methylation could be missed by their analytical approach.

Taken together, these results indicate that these three methods have complementary strengths in detecting DNA methylation in Arabidopsis.

## References

1. Jurka J, Klonowski P, Dagman V, Pelton P (1996) CENSOR--a program for identification and elimination of repetitive elements from DNA sequences. Comput Chem 20: 119-121.

2. Jurka J, Kapitonov VV, Pavlicek A, Klonowski P, Kohany O, et al. (2005) Repbase Update, a database of eukaryotic repetitive elements. Cytogenet Genome Res 110: 462-467.

3. Lippman Z, Gendrel AV, Black M, Vaughn MW, Dedhia N, et al. (2004) Role of transposable elements in heterochromatin and epigenetic control. Nature 430: 471-476.

4. Cervera MT, Ruiz-Garcia L, Martinez-Zapater JM (2002) Analysis of DNA methylation in Arabidopsis thaliana based on methylation-sensitive AFLP markers. Mol Genet Genomics 268: 543-552.

5. Lippman Z, Gendrel AV, Colot V, Martienssen R (2005) Profiling DNA methylation patterns using genomic tiling microarrays. Nat Methods 2: 219-224.

6. Schumacher A, Kapranov P, Kaminsky Z, Flanagan J, Assadzadeh A, et al. (2006) Microarray-based DNA methylation profiling: technology and applications. Nucleic Acids Res 34: 528-542.

7. Dudoit S, Yang YH, Callow MJ, Speed TP (2002) Statistical methods for identifying differentially expressed genes in replicated cDNA microarray experiments. *Statistica Sinica* 12: 111-140.

8. Cleveland WS, Grosse E (1991) Computational Methods for Local Fitting. *Statistics and Computing* 1: 47-92.

9. Martienssen RA, Doerge RW, Colot V (2005) Epigenomic mapping in Arabidopsis using tiling microarrays. Chromosome Res 13: 299-308.

10. Craig BA, Black MA, Doerge RW (2003) Gene expression data: The technology and statistical analysis. Journal of Agricultural Biological and Environmental Statistics 8: 1-28.

11. Zhang X, Yazaki J, Sundaresan A, Cokus S, Chan SW, et al. (2006) Genome-wide high-resolution mapping and functional analysis of DNA methylation in arabidopsis. Cell 126: 1189-1201.

12. Zilberman D, Gehring M, Tran RK, Ballinger T, Henikoff S (2007) Genome-wide analysis of Arabidopsis thaliana DNA methylation uncovers an interdependence between methylation and transcription. Nat Genet 39: 61-69.

13. Rangwala SH, Elumalai R, Vanier C, Ozkan H, Galbraith DW, et al. (2006) Meiotically stable natural epialleles of Sadhu, a novel Arabidopsis retroposon. PLoS Genet 2: e36.

14. Tran RK, Henikoff JG, Zilberman D, Ditt RF, Jacobsen SE, et al. (2005) DNA methylation profiling identifies CG methylation clusters in Arabidopsis genes. Curr Biol 15: 154-159.
