# Supplementary material for: Epigenetic Natural Variation in Arabidopsis thaliana
Source: PLoS Biol. 2007 Jun 19;5(7):e174. doi: 10.1371/journal.pbio.0050174 (PMC1892575; doi:10.1371/journal.pbio.0050174)
Supplement: Supplementary file 7 [file pbio.0050174.st001.doc]

Supplemental Table 1: Genes with Ler-Col CNPs

| **Gene** | **Ler vs Col** | **Borevitz et al.** |
| --- | --- | --- |
| AT4G00140 | + |  |
| AT4G00420 | + |  |
| AT4G00440 | - |  |
| AT4G00460 | - |  |
| AT4G00510 | - |  |
| AT4G00520 | - |  |
| AT4G00610 | + |  |
| AT4G00730 | - |  |
| AT4G00750 | + |  |
| AT4G01000 | + |  |
| AT4G01010 | + |  |
| AT4G01220 | - |  |
| AT4G01340 | - |  |
| AT4G01490 | - |  |
| AT4G01595 | - |  |
| AT4G01630 | - |  |
| AT4G01640 | - |  |
| AT4G01750 | - |  |
| AT4G01810 | + |  |
| AT4G01860 | - |  |
| AT4G01883 | - |  |
| AT4G01890 | - |  |
| AT4G01897 | - |  |
| AT4G01900 | - |  |
| AT4G01910 | - | Yes |
| AT4G01915 | - |  |
| AT4G01920 | - | Yes |
| AT4G01925 | - |  |
| AT4G01930 | - |  |
| AT4G01935 | - |  |
| AT4G01950 | - |  |
| AT4G01975 | - |  |
| AT4G02070 | - |  |
| AT4G02130 | - |  |
| AT4G02150 | + |  |
| AT4G02320 | + |  |
| AT4G02360 | - | Yes |
| AT4G02370 | 0 | No |
| AT4G02400 | - |  |
| AT4G02490 | - | Yes |
| AT4G02540 | - |  |
| AT4G02710 | - |  |
| AT4G02725 | - |  |
| AT4G02830 | - |  |
| AT4G02910 | - |  |
| AT4G02930 | - |  |
| AT4G02960 | + |  |
| AT4G03050 | + |  |
| AT4G03060 | + |  |
| AT4G03070 | + |  |
| AT4G03080 | + |  |
| AT4G03090 | + |  |
| AT4G03292 | - |  |
| AT4G03300 | - |  |
| AT4G03310 | - |  |
| AT4G03380 | - |  |
| AT4G03470 | - | Yes |
| AT4G03480 | - | Yes |
| AT4G03490 | - | Yes |
| AT4G03500 | - | Yes |
| AT4G03510 | - | Yes |
| AT4G03520 | - |  |
| AT4G03750 | + |  |
| AT4G03770 | + |  |
| AT4G03800 | - | Yes |
| AT4G03810 | - |  |
| AT4G03840 | + |  |
| AT4G03850 | + |  |
| AT4G03870 | - |  |
| AT4G03913 | - |  |
| AT4G03920 | - |  |
| AT4G03981 | - |  |
| AT4G04000 | - | Yes |
| AT4G04150 | - |  |
| AT4G04165 | + |  |
| AT4G04167 | + |  |
| AT4G04200 | - |  |
| AT4G04250 | - |  |
| AT4G04280 | + |  |
| AT4G04330 | - |  |
| AT4G04380 | - |  |
| AT4G04390 | - | Yes |
| AT4G04392 | - |  |
| AT4G04393 | - |  |
| AT4G04395 | - |  |
| AT4G04400 | - |  |
| AT4G04402 | - |  |
| AT4G04404 | - |  |
| AT4G04500 | 0 | No |
| AT4G04510 | - |  |
| AT4G04590 | - |  |
| AT4G04635 | - |  |
| AT4G04655 | + |  |
| AT4G04700 | - |  |
| AT4G04740 | - |  |
| AT4G04780 | - |  |
| AT4G04790 | - |  |
| AT4G04800 | - |  |
| AT4G04830 | - |  |
| AT4G04850 | - |  |
| AT4G04955 | - |  |
| AT4G05030 | - |  |
| AT4G05040 | - |  |
| AT4G05073 | - |  |
| AT4G05133 | - |  |
| AT4G05170 | + |  |
| AT4G05270 | - |  |
| AT4G05280 | + |  |
| AT4G05300 | + |  |
| AT4G05310 | - |  |
| AT4G05450 | - |  |
| AT4G05490 | - |  |
| AT4G05510 | + |  |
| AT4G05520 | - |  |
| AT4G05523 | - |  |
| AT4G05526 | - |  |
| AT4G05553 | + |  |
| AT4G05592 | - |  |
| AT4G05631 | - |  |
| AT4G05633 | + |  |
| AT4G05635 | - |  |
| AT4G06196 | - |  |
| AT4G06479 | + |  |
| AT4G06480 | + |  |
| AT4G06490 | - |  |
| AT4G06495 | + |  |
| AT4G06496 | + |  |
| AT4G06506 | + |  |
| AT4G06517 | - |  |
| AT4G06532 | + |  |
| AT4G06566 | + |  |
| AT4G06572 | + |  |
| AT4G06582 | + |  |
| AT4G06602 | + |  |
| AT4G06603 | + |  |
| AT4G06604 | + |  |
| AT4G06628 | + |  |
| AT4G06656 | + |  |
| AT4G06666 | + |  |
| AT4G06670 | + |  |
| AT4G06698 | + |  |
| AT4G06700 | + |  |
| AT4G06714 | + |  |
| AT4G06750 | - |  |
| AT4G07031 | + |  |
| AT4G07310 | - |  |
| AT4G07331 | + |  |
| AT4G07410 | - |  |
| AT4G07415 | + |  |
| AT4G07425 | - |  |
| AT4G07454 | - |  |
| AT4G07480 | - |  |
| AT4G07495 | - |  |
| AT4G07510 | - |  |
| AT4G07516 | - |  |
| AT4G07520 | + |  |
| AT4G07521 | - |  |
| AT4G07524 | + |  |
| AT4G07580 | - |  |
| AT4G07662 | - |  |
| AT4G07666 | - |  |
| AT4G07670 | - |  |
| AT4G07675 | - |  |
| AT4G07696 | - |  |
| AT4G07700 | - |  |
| AT4G07706 | - |  |
| AT4G07713 | - |  |
| AT4G07720 | - |  |
| AT4G07738 | - |  |
| AT4G07740 | - |  |
| AT4G07770 | - | Yes |
| AT4G07795 | - |  |
| AT4G07800 | - |  |
| AT4G07803 | - |  |
| AT4G07806 | - |  |
| AT4G07810 | - |  |
| AT4G07825 | - |  |
| AT4G07840 | - |  |
| AT4G07843 | - |  |
| AT4G07868 | - |  |
| AT4G07874 | - |  |
| AT4G07915 | - |  |
| AT4G07932 | - |  |
| AT4G07935 | - |  |
| AT4G07938 | + |  |
| AT4G08033 | + |  |
| AT4G08039 | - |  |
| AT4G08050 | + |  |
| AT4G08078 | + |  |
| AT4G08080 | + |  |
| AT4G08090 | - |  |
| AT4G08094 | - |  |
| AT4G08095 | + |  |
| AT4G08100 | + |  |
| AT4G08101 | - |  |
| AT4G08106 | - |  |
| AT4G08111 | + |  |
| AT4G08170 | + |  |
| AT4G08190 | + |  |
| AT4G08340 | - |  |
| AT4G08375 | - |  |
| AT4G08480 | - |  |
| AT4G08540 | - |  |
| AT4G08550 | - |  |
| AT4G08680 | - |  |
| AT4G08710 | + |  |
| AT4G08750 | + |  |
| AT4G08760 | + |  |
| AT4G08868 | - |  |
| AT4G08874 | - |  |
| AT4G08995 | - |  |
| AT4G09146 | - |  |
| AT4G09200 | - |  |
| AT4G09205 | - |  |
| AT4G09210 | - |  |
| AT4G09220 | - |  |
| AT4G09230 | - |  |
| AT4G09250 | - |  |
| AT4G09255 | - |  |
| AT4G09260 | - |  |
| AT4G09280 | - |  |
| AT4G09300 | - |  |
| AT4G09310 | - |  |
| AT4G09313 | - |  |
| AT4G09316 | - |  |
| AT4G09320 | - |  |
| AT4G09360 | - |  |
| AT4G09390 | - |  |
| AT4G09400 | - |  |
| AT4G09410 | - |  |
| AT4G09420 | - |  |
| AT4G09425 | + |  |
| AT4G09430 | - |  |
| AT4G09450 | - |  |
| AT4G09455 | - |  |
| AT4G09480 | - |  |
| AT4G09565 | - |  |
| AT4G09595 | - |  |
| AT4G09660 | - |  |
| AT4G09690 | - | Yes |
| AT4G09700 | - |  |
| AT4G09710 | - | Yes |
| AT4G09720 | - |  |
| AT4G09750 | - |  |
| AT4G09775 | - |  |
| AT4G09780 | - | Partial |
| AT4G09900 | - |  |
| AT4G09930 | - |  |
| AT4G09940 | - |  |
| AT4G09950 | - |  |
| AT4G10080 | + |  |
| AT4G10140 | - |  |
| AT4G10310 | - |  |
| AT4G10340 | + |  |
| AT4G10350 | - |  |
| AT4G10450 | - |  |
| AT4G10460 | - |  |
| AT4G10550 | - |  |
| AT4G10570 | - |  |
| AT4G10600 | - |  |
| AT4G10640 | - |  |
| AT4G10650 | - |  |
| AT4G10690 | - |  |
| AT4G10695 | - |  |
| AT4G10700 | - |  |
| AT4G10770 | - |  |
| AT4G10780 | - | Yes |
| AT4G10830 | - |  |
| AT4G10890 | - |  |
| AT4G11030 | - |  |
| AT4G11070 | - |  |
| AT4G11080 | - |  |
| AT4G11190 | - |  |
| AT4G11200 | - |  |
| AT4G11375 | + |  |
| AT4G11460 | - |  |
| AT4G11490 | - |  |
| AT4G11570 | - |  |
| AT4G11630 | - |  |
| AT4G11780 | - |  |
| AT4G11800 | - |  |
| AT4G11850 | - |  |
| AT4G11910 | - |  |
| AT4G11960 | - |  |
| AT4G11970 | - |  |
| AT4G11980 | - |  |
| AT4G12030 | - |  |
| AT4G12060 | - |  |
| AT4G12120 | - |  |
| AT4G12180 | - | Yes |
| AT4G12275 | - |  |
| AT4G12440 | + |  |
| AT4G12460 | - |  |
| AT4G12830 | - |  |
| AT4G12860 | - |  |
| AT4G13010 | - |  |
| AT4G13020 | - |  |
| AT4G13050 | - |  |
| AT4G13070 | - |  |
| AT4G13110 | - |  |
| AT4G13160 | - |  |
| AT4G13180 | - |  |
| AT4G13230 | + |  |
| AT4G13253 | - |  |
| AT4G13255 | - |  |
| AT4G13258 | - |  |
| AT4G13259 | - |  |
| AT4G13300 | - |  |
| AT4G13330 | - |  |
| AT4G13345 | - |  |
| AT4G13430 | + |  |
| AT4G13442 | - |  |
| AT4G13510 | + |  |
| AT4G13630 | - |  |
| AT4G13640 | - |  |
| AT4G13690 | - |  |
| AT4G13700 | - |  |
| AT4G13710 | + |  |
| AT4G13800 | - |  |
| AT4G13885 | - |  |
| AT4G13900 | - |  |
| AT4G14070 | - |  |
| AT4G14147 | - |  |
| AT4G14370 | - |  |
| AT4G14385 | - |  |
| AT4G14400 | 0 | No |
| AT4G14460 | - |  |
| AT4G14470 | - | Yes |
| AT4G14730 | - |  |
| AT4G14805 | + |  |
| AT4G15090 | + |  |
| AT4G15096 | - |  |
| AT4G15100 | + |  |
| AT4G15215 | - |  |
| AT4G15236 | - |  |
| AT4G15290 | - |  |
| AT4G15310 | - |  |
| AT4G15320 | + |  |
| AT4G15340 | - |  |
| AT4G15410 | + |  |
| AT4G15420 | - |  |
| AT4G15570 | - | Partial |
| AT4G15590 | - | Yes |
| AT4G15700 | - |  |
| AT4G15720 | - |  |
| AT4G15930 | - |  |
| AT4G15940 | - |  |
| AT4G15950 | - |  |
| AT4G16000 | + |  |
| AT4G16010 | - |  |
| AT4G16015 | - |  |
| AT4G16020 | - |  |
| AT4G16180 | - |  |
| AT4G16230 | - |  |
| AT4G16280 | + |  |
| AT4G16490 | + |  |
| AT4G16720 | - |  |
| AT4G16760 | - |  |
| AT4G16765 | - |  |
| AT4G16770 | - |  |
| AT4G16800 | - |  |
| AT4G16810 | - |  |
| AT4G16860 | - |  |
| AT4G16870 | + |  |
| AT4G16890 | - |  |
| AT4G16910 | + |  |
| AT4G16920 | - |  |
| AT4G16930 | - |  |
| AT4G16935 | - |  |
| AT4G16940 | - |  |
| AT4G16960 | - |  |
| AT4G16970 | + |  |
| AT4G17070 | - |  |
| AT4G17240 | + |  |
| AT4G17410 | + |  |
| AT4G17450 | - |  |
| AT4G17580 | + |  |
| AT4G17910 | + |  |
| AT4G18090 | - |  |
| AT4G18250 | - |  |
| AT4G18290 | - |  |
| AT4G18310 | - |  |
| AT4G18410 | - | Yes |
| AT4G18420 | - |  |
| AT4G18430 | - |  |
| AT4G18580 | - |  |
| AT4G18590 | - |  |
| AT4G18730 | - |  |
| AT4G18860 | - |  |
| AT4G19038 | - |  |
| AT4G19120 | - | Yes |
| AT4G19240 | + |  |
| AT4G19410 | - |  |
| AT4G19440 | - |  |
| AT4G19470 | - |  |
| AT4G19490 | - |  |
| AT4G19500 | - |  |
| AT4G19510 | - | Yes |
| AT4G19550 | - |  |
| AT4G19650 | - |  |
| AT4G19780 | - |  |
| AT4G19790 | - |  |
| AT4G19930 | - |  |
| AT4G19960 | - |  |
| AT4G19990 | - | Yes |
| AT4G20040 | - |  |
| AT4G20110 | 0 | No |
| AT4G20140 | + |  |
| AT4G20170 | - |  |
| AT4G20200 | - |  |
| AT4G20210 | - | Yes |
| AT4G20220 | - |  |
| AT4G20230 | - |  |
| AT4G20240 | - |  |
| AT4G20250 | - |  |
| AT4G20320 | - |  |
| AT4G20350 | - |  |
| AT4G20365 | - |  |
| AT4G20440 | - |  |
| AT4G20490 | - |  |
| AT4G20520 | - |  |
| AT4G20530 | - |  |
| AT4G20540 | - |  |
| AT4G20550 | - |  |
| AT4G20560 | - |  |
| AT4G20570 | - |  |
| AT4G20580 | - |  |
| AT4G20590 | - |  |
| AT4G20600 | - |  |
| AT4G20610 | - |  |
| AT4G20620 | - |  |
| AT4G20630 | - |  |
| AT4G20640 | - |  |
| AT4G20650 | - |  |
| AT4G20670 | - |  |
| AT4G20680 | - |  |
| AT4G20700 | - |  |
| AT4G20710 | - |  |
| AT4G20860 | 0 | No |
| AT4G21110 | - |  |
| AT4G21190 | - |  |
| AT4G21380 | + |  |
| AT4G21400 | - |  |
| AT4G21420 | - |  |
| AT4G21640 | + |  |
| AT4G21865 | - |  |
| AT4G22040 | - |  |
| AT4G22110 | - |  |
| AT4G22120 | - |  |
| AT4G22180 | + |  |
| AT4G22310 | - |  |
| AT4G22320 | - |  |
| AT4G22390 | + |  |
| AT4G22415 | + |  |
| AT4G22450 | - |  |
| AT4G22690 | - |  |
| AT4G22870 | - |  |
| AT4G23120 | - |  |
| AT4G23160 | - |  |
| AT4G23220 | - |  |
| AT4G23230 | - |  |
| AT4G23240 | - |  |
| AT4G23250 | 0 | No |
| AT4G23620 | - |  |
| AT4G23820 | - |  |
| AT4G23860 | - |  |
| AT4G24980 | - |  |
| AT4G25315 | - |  |
| AT4G25420 | - |  |
| AT4G25450 | - |  |
| AT4G25640 | 0 | No |
| AT4G25870 | - |  |
| AT4G26190 | - |  |
| AT4G26360 | - |  |
| AT4G27040 | + |  |
| AT4G27050 | + |  |
| AT4G27080 | + |  |
| AT4G27190 | - | Yes |
| AT4G27200 | - |  |
| AT4G27210 | - |  |
| AT4G27220 | - | Yes |
| AT4G27745 | + |  |
| AT4G27830 | - |  |
| AT4G27850 | - |  |
| AT4G27870 | - |  |
| AT4G28410 | + |  |
| AT4G28420 | 0 | No |
| AT4G28650 | + |  |
| AT4G28780 | + |  |
| AT4G29770 | - |  |
| AT4G29960 | - |  |
| AT4G30200 | - |  |
| AT4G30935 | + |  |
| AT4G30940 | + |  |
| AT4G30950 | + |  |
| AT4G31120 | - |  |
| AT4G31140 | + |  |
| AT4G31880 | + |  |
| AT4G32080 | + |  |
| AT4G32210 | - |  |
| AT4G33290 | + |  |
| AT4G33300 | + |  |
| AT4G33390 | + |  |
| AT4G33490 | + |  |
| AT4G33905 | - |  |
| AT4G34650 | - |  |
| AT4G36120 | - |  |
| AT4G36140 | - |  |
| AT4G36150 | - |  |
| AT4G37235 | + |  |
| AT4G37620 | - |  |
| AT4G38170 | + |  |
| AT4G38750 | + |  |
| AT4G38780 | + |  |
| AT4G39060 | + |  |
| AT4G39930 | - |  |
| AT4G39940 | - |  |
