# Supplementary material for: Epigenetic Natural Variation in Arabidopsis thaliana
Source: PLoS Biol. 2007 Jun 19;5(7):e174. doi: 10.1371/journal.pbio.0050174 (PMC1892575; doi:10.1371/journal.pbio.0050174)
Supplement: Supplementary file 8 [file pbio.0050174.st002.doc]

Supplemental Table 2: PCR-based validation of microarray results

|  | **Col-0** |  |  |  | **Ler** |  |
| --- | --- | --- | --- | --- | --- | --- |
|  | *McrBC-PCR* | *Array* | *Array* | *Array* | *McrBC-PCR* | *Array* |
| **Feature** | **Vaughn** | **Vaughn** | **Zhang** | **Zilberman** | **Vaughn** | **Vaughn** |
| Ta03h08 | **+** | **-** | **+** | **+** | **+** | **+** |
| Ta07h02 | **+** | **-** | **-** | **-** | **+** | **+** |
| Ta17d08 | **+** | **+** | **+** | **+** | **+** | **+** |
| Ta31g09 | **+** | **+** | **-** | **+** | **-** | **-** |
| Ta71d10 | **+** | **+** | **-** | **+** | **+** | **+** |
| Ta83c04 | **+** | **+** | **-** | **-** | **+** | **+** |
| Ta86g01 | **+** | **-** | **+** | **+** | **-** | **-** |
| Tb12e04 | **+** | **+** | **-** | **+** | **-** | **-** |
| Tb17d03 | **+** | **-** | **+** | **+** | **+** | **+** |
| Tb23a01 | **+** | **+** | **-** | **+** | **-** | **-** |
| Tb33d12 | **+** | **+** | **+** | **-** | **-** | **-** |
| Tb39g10 | **+** | **+** | **+** | **+** | **+** | **+** |
| Tb45c04 | **+** | **+** | **+** | **-** | **-** | **-** |
| Tb54f11 | **+** | **+** | **+** | **+** | **-** | **-** |
| Tb63b02 | **+** | **+** | **-** | **+** | **-** | **-** |
| Tb73g06 | **+** | **+** | **+** | **-** | **-** | **-** |
| Tb97d02 | **+** | **+** | **-** | **-** | **-** | **-** |
| Tb98h02 | **+** | **+** | **-** | **-** | **-** | **-** |
| Ta10g04 | **-** | **-** | **-** | **-** | **+** | **+** |
| Ta10g05 | **-** | **-** | **-** | **-** | **+** | **+** |
| Ta13a01 | **-** | **+** | **-** | **+** | **-** | **-** |
| Ta13d11 | **-** | **-** | **-** | **-** | **+** | **+** |
| Ta17f10 | **-** | **-** | **-** | **-** | **+** | **+** |
| Ta33d12 | **-** | **-** | **-** | **-** | **+** | **+** |
| Ta51d07 | **-** | **-** | **-** | **-** | **+** | **+** |
| Tb28c06 | **-** | **-** | **-** | **-** | **+** | **+** |
| Tb57h12 | **-** | **-** | **-** | **-** | **+** | **+** |
| Tc00c06 | **-** | **+** | **+** | **+** | **-** | **-** |
