# Supplementary material for: Epigenetic Natural Variation in Arabidopsis thaliana
Source: PLoS Biol. 2007 Jun 19;5(7):e174. doi: 10.1371/journal.pbio.0050174 (PMC1892575; doi:10.1371/journal.pbio.0050174)
Supplement: Supplementary file 9 [file pbio.0050174.st003.doc]

|  | **At4g28850** | **At4g18020** |
| --- | --- | --- |
| **F1** | Col x Ler  8/8 Have Col-0 5mC | Col x Ler  7/8 Have Col-0 5mC |
| **F2** | Col 8 (8):23 (23):9 (0) Ler | Col 11 (11):17 (15): 9 (0) Ler |
| **F1** | Ler x Col  7/8 Have Col-0 5mC | Ler x Col  7/8 Have Col-0 5mC |
| **F2** | Col 8 (5):23 (18):9 (0) Ler | Col 7 (7):24 (23): 7 (0) Ler |

**Supplemental Table 3: Summary of methylation heritability studies**
