# Supplementary material for: Epigenetic Natural Variation in Arabidopsis thaliana
Source: PLoS Biol. 2007 Jun 19;5(7):e174. doi: 10.1371/journal.pbio.0050174 (PMC1892575; doi:10.1371/journal.pbio.0050174)
Supplement: Supplementary file 10 [file pbio.0050174.st004.doc]

**Supplemental Table 4: Correlation between Ler CNP and expression levels**

|  | *No CNP* | *Decrease Ler* | *Decrease Col* |
| --- | --- | --- | --- |
| Unexpressed | 756 | 38 (21) | ND |
| No Difference | 2423 | 46 (65) | ND |
| Up, Ler v Col | 55 | 1 (1) | ND |
| Up, Col v Ler | 58 | 3 (1) | ND |
| Total | 3292 | 88 | ND |
