# Supplementary material for: Epigenetic Natural Variation in Arabidopsis thaliana
Source: PLoS Biol. 2007 Jun 19;5(7):e174. doi: 10.1371/journal.pbio.0050174 (PMC1892575; doi:10.1371/journal.pbio.0050174)
Supplement: Supplementary file 11 [file pbio.0050174.st005.doc]

**Supplemental Table 5: Methylation polymorphism versus gene expression**

*Expression*

*Methylation*

|  | *None* | *Both* | *Col only* | *Ler only* |
| --- | --- | --- | --- | --- |
| Unexpressed | 289 | 59 (131) | 29 (37) | 44 (51) |
| No Difference | 822 | 447 (374) | 112 (106) | 145 (144) |
| Down, Col vs Ler | 14 | 7 (6) | 4 (2) | 6 (2) |
| Up, Col vs Ler | 19 | 7 (9) | 3 (2) | 6 (3) |
| Total | 1144 | 520 | 148 | 169 |
