# Supplementary material for: Epigenetic Natural Variation in Arabidopsis thaliana
Source: PLoS Biol. 2007 Jun 19;5(7):e174. doi: 10.1371/journal.pbio.0050174 (PMC1892575; doi:10.1371/journal.pbio.0050174)
Supplement: Supplementary file 12 [file pbio.0050174.st006.doc]

**Supplemental Table 6: Unannotated control tiles used in statistical analysis of methylation microarray data**

ta82e05

ta81e03

ta92f07

ta65a03

ta86e06

ta84d07

ta88d09

ta86e02

ta70e09

ta82c09

ta70h03

ta94d04

ta91h05

ta96c08

ta64b08

ta85f11

ta97d11

ta84d04

ta76c05

ta74d09

ta66b03

ta86g02

ta80c10

ta80d10

ta68h12

ta72h01

ta95a12

ta92c11

ta88c03

ta97d05

ta94a01

ta93c09

ta82h06

ta86d02

ta65c03

ta92d09

ta66b07

ta80g09

ta68e04

ta84e02

ta72g06

ta68c02

ta66c02

ta70h09

ta77e08

ta91e07

ta93d04

ta93a05

ta91g05

ta89f09

ta84e06

ta64d07

ta92e04

ta66h04

ta93d08

ta76c11

ta87h07

ta64b07

ta86a03

ta69c01

ta90a02

ta64d06

ta89e05

ta80f03

ta88d03

ta66d10

ta97d10

ta91h02

ta88e03

ta72a06

ta80c11

ta92e03

ta73d07

ta86b07

ta89f07

ta94a02

ta84g02

ta85f07

ta94h05

ta84f06

ta92e01

ta88b04

ta67a09

ta84c04

ta74c06

ta93f10

ta71b06

ta92c07

ta80a05

ta69c02

ta91b04

ta69d02

ta87h10

ta78e09

ta75a07

ta64b03

ta70g10

ta89h01

ta76c07

ta80g12

ta80b04

ta68a05

ta84e05

ta75g12

ta79h09

ta91h07

ta80d09

ta89e04

ta67h09

ta78e08

ta93c10

ta83c08

ta93h12

ta80a06

ta92c08

ta73d06

ta84e04

ta67h02

ta83d09

ta95c01

ta84g12

ta97a06

ta69a10

ta84g03

ta79f03

ta86g11

ta85f06

ta92g06

ta72h11

ta86a01

ta92c06

ta72g05

ta82e12

ta80a04

ta93h11

ta76c09

ta79g04

ta68e06

ta96f06

ta76h05

ta95h04

ta64c03

ta67a06

ta77d02

ta96d12

ta70f01

ta93f01

ta84f12

ta69b12

ta74b12

ta87h09

ta70e03

ta88g08

ta65h01

ta72h02

ta95f02

ta80g08

ta80a03

ta88g01

ta85h03

ta78e10

ta92c12

ta80g03

ta93g04

ta67d02

ta74c04

ta67b10

ta79d08

ta73h05

ta77a11

ta84f03

ta93e11

ta85d01

ta86e07

ta71a05

ta68g10

ta67e01

ta77h05

ta86d08

ta66f03

ta88d01

ta95b06

ta81e11

ta73e01

ta86h02

ta85f10

ta74d01

ta93e08

ta69c07

ta88h05

ta92h06

ta93g06

ta80g02

ta85h04

ta81c03

ta84d03

ta84h06

ta91h09

ta70g01

ta75d02

ta73h01

ta88b05

ta75d01

ta89e06

ta93f02

ta74d06

ta91d09

ta88c02

ta92e02

ta93h07

ta80d02

ta82b10

ta93f07

ta73c01

ta72a01

ta85g06

ta69g04

ta83d10

ta71c05

ta89f08

ta94b08

ta78d08

ta97g04

ta95g09

ta97f08

ta78f12

ta64b06

ta86c01

ta94d03

ta73d12

ta86g12

ta87b09

ta93c11

ta67d11

ta67a10

ta88g02

tb80d05

tb08c12

tb54e07

ta88h06

tb07d11

ta87f08

tb92a11

tb87e06

tb66f12

tb76e02

tb87d06

tb67a01

tb84e09

tb60h09

tb94g11

tc05g12

tb74b05

tb87h02

tc09f03

tb10e02

tb86b02

ta82a12

tb68f07

tb27b11

tb44b04

ta90d03

tb07c09

tb94c09

tb52b11

tb33h12

tb48a10

tc11e05

tb00f03

tc11a02

tc12b10

tb96g06

tb92a12

tb87e04

tb22e01

tb41f12

tb60b05

tb78f07

tb60f06

tb75c09

tb54a12

tb38a08

tb85d09

tb97b07

ta88f07

tc02a01

tb75f09

tb65e08

tb33b10

tb72a11

tb35e05

tb22h01

ta90a09

ta95g08

tb55c06

ta98g03

ta78g02

tb42c03

tc06d05

tb09b05

tb15d03

tc13c06

tb95a02

tb19d06

tb67e08

ta80f07

tc05e03

tb82b03

ta94b02

tb48d11

tc08h04

tb05c10

tb50e06

tc06c08

ta94f10

ta88a01

tb41c06

tb80c09

tb54c07

tb98g11

tb91c02

tb54b03

tb91g03

tb50b11

tc13a04

tb88c12

tb79f12

tb92h01

ta91e01

tb67e12

tb16b05

ta84e09

tc04b03

tb49c07

tb04f09

ta77b10

tb88h03

tc06e10

tb30g10

tb44h02

tc06h02

ta81f12

ta85e10

tc08e06

tb76e04

ta86a06

tb60g06

tb06a02

tb60a11

tb47g09

tb56h01

ta83b01

tb80g10

tb28c12

tb79d06

tb12g04

tb58e06

tb74b12

tb01g04

tb59g03

tb05c06

tb87g12

tb99h11

ta92a05

tb45e10

ta88g12

tb40d12

tb10f09

tb88e01

tb71d04

tb60c03

tb14b07

tb97f02

tb64g03

tb94d10

tb61e04

tb33g06

tb51e06

tb83d03

tb71c09

tb50a06

tc13a10

tb42c11

tb94h02

tb65a02

tb44b12

tb64d03

tb49g05

tc10c02

tb90b02

tb89g06

tb20c11

tb45c12

tb34h09

ta83b11

ta82a07

tb03a11

ta78b07

tb82b02

tb55b04

tc06d09

tb61e08

tb94e04

ta78g09

ta79g09

tc06g04

ta87g11

tb16c02

tb05b12

tb03h07

ta91d12

tb78h08

tb44b08

tb68b07

tb51d08

tc12g03

tb48f08

tb64e10

tb84c10

tb54c08

tb50e01

tb07e11

tb33h10

tb62f04

ta99e06

tb54h05

ta87c11

tb54g03

tb64h10

tb76b03

tb08h10

ta90h12

tb08d04

tb98f03

tb16c05

ta92d06

tb50f08

tb41g01

tb92g08

tb58b08

tb29c08

tb81b06

tb58d07

tb47f06

tb52g07

tb92f02

tb08b11

tb77b05

tc07d02

tb52e01

tb46e09

tc06b04

tc00c05

ta85b01

tb42e10

tb31a01

tb58g10

tc01h11

tb92g04

tb52d11

tb07e10

tb80g06

tb29h05

ta85e12

tb85d07

tb17c01

tb02g02

tb31f08

tc05c05

ta76a10

tb79g06

tb40e05

tb50h02

tb97c06

tb77g02

ta92c04

tb22h08

tb57b10

tb40c02

tb74b04

tb43h06

tc00c08

tb69a10

tb96f10

tb71f05

tb91a06

tb53c10

tb29b10

tb42h04

tb02c07

tb79g01

ta88h01

tb84e07

tb29e05

tb26d07

tb54d02

tb73f07

tc12f12

tb24d07

tb95b02

ta83a12

tb75c12

tc12a09

tb26a12

ta90d05

tb50g08

tb92b01

tb29c11

tb50d12

tb97h09

tb78g09

tb03h11

tb36b05

ta89g07

tb86e11

ta87f09

tb58b03

tb49f11

tb99a11

tb59e12

ta88f08

tb94a04

tb58c09

ta89h08

ta81g01

tb67g08

tb02a10

tb55h12

tb02g01

tb25f11

tb62e06

tb24d06

tb45h04

tc12g04

tb88f10

ta99e01

ta98e08

ta84h08

tb77b04

tb80d01

tb68a06

tb53e12

tb58b02

ta79c12

tb44a09

tb42e09

tb61c06

tb30h07

tb43a04

ta76b07

tb86g03

tb07e12

tb35d12

tb49h03

tb40b09

tb14g06

tb64h01

tb36e06

tb72b12

tb05b04

tb98g10

tb88c10

tb99d07

ta92e10

tb45g03

tb53h12

tb57a03

tc11e04

tb52g09

tb14g04

tb67d02

ta77f04

tb16e05

tb95f02

tb63g05

tb61h07
