# Supplementary material for: Epigenetic Natural Variation in Arabidopsis thaliana
Source: PLoS Biol. 2007 Jun 19;5(7):e174. doi: 10.1371/journal.pbio.0050174 (PMC1892575; doi:10.1371/journal.pbio.0050174)
Supplement: Supplementary file 13 [file pbio.0050174.st007.doc]

Supplemental Table 7: Primer sequences used for McrBC PCR reactions

| **Primer Name** | **Primer Sequence(5’-3’)** |
| --- | --- |
| tb63b02F | ATATAGGTGGTTCGTCGATGG |
| tb63b02R | AGATGAGACAATAGGGGCATT |
| At4g14570_F1 | GAATTGCATGTTGGTTTGGA |
| At4g14570_R1 | CGAAATATGACCCGACAACA |
| At4g14570_F2 | TGGATGGTATGGCACTTTGT |
| At4g14570_R2 | CTTCTCCCCATTCCTCTTCC |
| At4g14570_F3 | CATAAGCAGCGGGTTTTGTC |
| At4g14570_R3 | TTTGCCATCTTTGCTGTGAG |
| At4g14570_F4 | TTGTTTCCCTGGGCTCTATG |
| At4g14570_R4 | GCAAGAGCGTTCCATGAATA |
| At4g14570_F5 | CCGGTGAGTGTTCCTGAAAT |
| 4g14570_R5 | TTACCTTCGGCAAGACCTTC, |
| 4g14570_F6 | TGCAGTCTCTACCTGGCAAA, |
| 4g14570_R6 | CAAATTTATCCGGGGCCTAT. |
