# Supplementary material for: Epigenetic Natural Variation in Arabidopsis thaliana
Source: PLoS Biol. 2007 Jun 19;5(7):e174. doi: 10.1371/journal.pbio.0050174 (PMC1892575; doi:10.1371/journal.pbio.0050174)
Supplement: Supplementary file 14 [file pbio.0050174.st008.doc]

**Supplemental Table 8: Comparison of our microarray results to other recent methylation profiling datasets**

| **Annotation Class** | **Tiles** | **Vaughn** | **Zhang*** | **Zilberman*** |
| --- | --- | --- | --- | --- |
| Gene | 9351 | 1991 | 1280 | 1823 |
| Gene+Repeat | 3694 | 908 | 660 | 3132 |
| Repeat (Tandem) | 645 | 162 | 131 | 272 |
| Retrotransposon | 2785 | 2066 | 1852 | 2562 |
| Transposon | 1766 | 975 | 868 | 1181 |
| Unannotated | 3105 | 399 | 315 | 479 |
| *Total* | *21346* | *6501* | *5106* | *9449* |
