# Supplementary material for: Epigenetic Natural Variation in Arabidopsis thaliana
Source: PLoS Biol. 2007 Jun 19;5(7):e174. doi: 10.1371/journal.pbio.0050174 (PMC1892575; doi:10.1371/journal.pbio.0050174)
Supplement: Supplementary file 15 [file pbio.0050174.st009.doc]

**Supplemental Table 9: Annotation of microarray features profiled in 96 natural variation accessions**

| **Feature** | **Gene** | **Annotation** |
| --- | --- | --- |
| ta03h08 | AT4G00500 | lipase class 3 family protein / calmodulin-binding heat-shock protein-related |
| ta07h02 | AT4G01310 | ribosomal protein L5 family protein |
| ta10g04 | AT4G01760 | DC1 domain-containing protein, near Class II TE |
| ta31g09 | AT4G04750 | sugar transporter family protein |
| ta71d10 | AT4G10770 | oligopeptide transporter OPT family protein |
| ta83c04 | AT4G13060 | F-box family protein |
| ta86g01 | AT4G13610 | DNA (cytosine-5-)-methyltransferase, near repeats |
| tb12e04 | AT4G18020 | pseudo-response regulator 2 (APRR2/TOC2) |
| tb17d03 | AT4G19020 | chromomethylase |
| tb28c06 | AT4G21320 | (2R)-phospho-3-sulfolactate synthase |
| tb33d12 | AT4G22240 | plastid-lipid associated protein |
| tb39g10 | AT4G23560 | glycosyl hydrolase family 9 protein |
| fwa | AT4G25530 | homeodomain protein FWA |
| tb54f11 | AT4G26960 | expressed protein |
| tb57h12 | AT4G27650 | pelota (PEL1) |
| tb63b02 | AT4G28850 | xyloglucosyl transferase |
| tb73g06 | AT4G31150 | endonuclease V family protein |
| tb98h02 | AT4G36780 | brassinosteroid signalling positive regulator-related |
